# Spontaneous Oxidation of Nitrous Acid to Nitric Acid in Supermicron Aqueous Droplets Is Acid-Accelerated

**DOI:** 10.1021/acsearthspacechem.5c00014

**Published:** 2025-04-21

**Authors:** Luke W. Monroe, Jack W. Hall, Graham M. Thornhill, Ryan C. Sullivan

**Affiliations:** † Department of Chemistry, Carnegie Mellon University, Pittsburgh, Pennsylvania 15213, United States; ‡ Department of Mechanical Engineering, Carnegie Mellon University, Pittsburgh, Pennsylvania 15213, United States

**Keywords:** atmospheric chemistry, particulate matter, chemical kinetics, microdroplet-accelerated chemistry, Raman spectroscopy

## Abstract

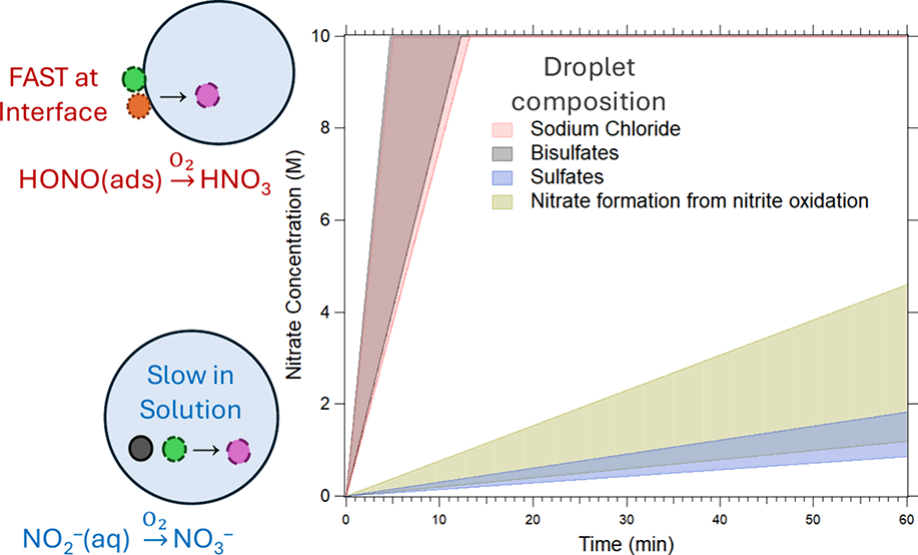

It is generally assumed that acidic submicron atmospheric aerosol particles do not constitute a significant sink for nitrous acid (HONO), as this weak acid would remain protonated and volatile, yet the uptake of HONO to larger less acidic particles is unexplored. Experiments on optically tweezed aerosol did not observe HONO gas uptake but instead revealed rapid oxidation of HONO to HNO_3_ in droplets of initial pH between 0 and 7.75. This oxidation was spontaneous at room temperature with no oxidant added and occurred over a subminute time scale. The reaction is accelerated under acidic conditions of pH < 2. We hypothesize that protonated HONO is restricted to the interfacial region while NO_2_^–^ is not, and HONO is therefore oxidized to HNO_3_ following a second-order rate dependence on the HONO concentration. The oxidation of HONO can thus be self-catalyzed in weakly buffered aerosol. Less acidic droplets (pH > 5.0) displayed an approximately 2 orders of magnitude lower conversion rate of HONO to HNO_3_, likely due to deprotonation and then slower oxidation of NO_2_^–^ directly to NO_3_^–^ with a first-order dependence on the NO_2_^–^ concentration. Production of HNO_3_ can drive a liquid–liquid phase separation of secondary organic aerosol, but an organic shell phase did not prevent oxidation of HONO to HNO_3_. This rapid conversion of HONO to HNO_3_ at the droplet interface due to an acidity-based transition in the reaction mechanism could represent a significant new sink for HONO and a source of strong inorganic acids in the atmosphere that are more readily removed through deposition.

## Introduction

Nitrous acid (HONO) in the atmosphere is an essential component of the nitrogen oxide chemical family that is involved in important reactions that cycle and regenerate atmospheric oxidants and drive ozone production. HONO is primarily present in polluted nighttime atmospheres due to its rapid photolysis to NO + OH but is also present at lower levels in remote environments and during the day where it continues to participate in chemical reactions.^1−3^

The multiphase chemical mechanisms that produce HONO in the environment have been an area of active research for more than 40 years.^4−17^ Models continue to underpredict tropospheric HONO concentrations, leading to a consensus that the current sources of HONO are inadequate due either to underestimation of their production rate or to currently unknown production pathways.^7,10,12,18,19^ The major identified HONO production pathways are the heterogeneous reaction of NO_2_ with water while absorbed on a surface or in a particle and the gas-phase reaction of NO + OH during the day.^1,6,12,20^ A growing focus has been the renoxification of NO_3_^–^ to NO_2_^–^/HONO on surfaces from photolysis, leading to constant regeneration of HONO during daylight hours that is quickly photolyzed.^13,15,17,21−27^

The chemical sink pathways of HONO have received less attention. Aside from daytime photolysis of HONO, other leading loss mechanisms are the partitioning of nitrite to aqueous media, reaction with the hydroxyl radical^1^ or hydrochloric acid,^28,29^ and dry deposition.^1−3,30^ NO_2_^–^ is difficult to measure reliably in collected aerosol particles, as steam-based aerosol collectors will convert NO_2_^–^ to NO_3_^–^.^18^

One common sink that is excluded when estimating the HONO loss rate is the contribution that aerosol particles make to HONO removal. Aerosol particles are usually the largest available surface area on which vapor condensation or heterogeneous reactions can happen. The p*K*_a_ of HONO is 3.2. Fine-mode aerosol particles are generally acidic due to the accumulation of strong inorganic acids such as H_2_SO_4_ and HNO_3_. Thus, submicron aerosols are assumed to not present a sink for HONO due to their high acidity that keeps HONO protonated and volatile. Most studies on aerosol sinks for HONO have focused on large dew/fog/cloud/rain droplets that are much more dilute and sufficiently less acidic, allowing for the deprotonation and greater gas-to-particle partitioning of HONO.^31−33^ Estimates place the deposition of HONO to these sources at 70–80% of HONO loss during relevant nocturnal conditions.^32^

After the deprotonation of HONO in the droplet to form NO_2_^–^, several reactions are known to occur that can affect the chemical balance of HONO and nitrogen oxides. Strong oxidants, such as ozone, hydroxyl radical, and hydrogen peroxide, can readily convert NO_2_^–^ to NO_3_^–^. Little consideration has been given to nitrite oxidation by O_2_ in the particle phase, with the exception that it may be accelerated during the water freezing process.^34,35^

This study utilizes aerosol optical tweezers (AOT) to levitate aqueous supermicron droplets and obtain the cavity-enhanced Raman spectrum of the droplet in real time. Analysis of the Raman spectrum provides information regarding changes in the chemical composition, size, refractive index, phase separations, and resulting morphology of the droplet. We present and discuss two sets of experiments below. Droplets were exposed to HONO vapor to determine the uptake rate by observing changes in the droplet composition using Raman spectroscopy in real time. Sodium nitrite droplets were monitored to observe the oxidation rate of NO_2_^–^ to NO_3_^–^ under nitrogen gas and air. The rate of HONO uptake is compared across droplets composed of differing inorganic ions and pH. The second set of experiments used droplets containing organic acids and secondary organic aerosol produced through terpene oxidation in the tweezers chamber. In all cases HONO vapor was found to partition to the aerosol droplet due to its varying rates of conversion to HNO_3_/NO_3_^–^; HONO/NO_2_^–^ was not observed in the droplet. The ability to reliably distinguish between and quantify NO_2_^–^ and NO_3_^–^ using real-time aerosol Raman spectroscopy enabled these novel observations of the spontaneous oxidation of HONO to HNO_3_ in droplets.

## Experimental Methods

### Chamber Design

The custom aerosol optical tweezers (AOT) system used is described elsewhere in detail, and a schematic of the AOT chamber from Gorkoswki et al. is included as Figure S1.^36−38^ The AOT consists of two chambers. The upper chamber is a gas mixing chamber, and the lower chamber is a trapping chamber. Aerosol is nebulized from a medical nebulizer (PARI TREK S) and drawn into the trapping chamber of the AOT. Droplets (typically greater than 4 μm diameter) are trapped in a gradient force trap resulting from a 532 nm laser at 0.06 W power focused via a 100× (NA 1.25) oil immersion objective. The backscattered Stokes-shifted Raman signal is collected through this same objective and is directed into a Raman spectrometer with a CCD camera (Princeton Instruments).

Conditioned air flow enters the gas mixing chamber before it flows into the trapping chamber and then vacates through a vacuum port. Total flow in was 0.3 lpm, and total vacuum flow out was 0.25 lpm. This slight positive pressure flow difference minimized contamination from the room air. The conditioned air in is split into three flows: dry air, humid air, and HONO_(g)_ flow. The HONO_(g)_ flow also passes through an aqueous solution, and the sum humid flow was set at 0.21 lpm between the HONO and humid air flows while dry air was 0.09 lpm. This maintained relative humidity (RH) at 70–80% for all experiments. All experiments were conducted at a lab temperature of 20 °C.

HONO_(g)_ was generated by using air passed through a gas washer containing 0.3 M NaNO_2_ acidified with H_2_SO_4_. The airflow through the gas washer was maintained between 0.02 and 0.06 lpm, resulting in 46 ± 13 to 140 ± 37 ppm_v_ HONO_(g)_. This flow rate is near the limit of airflow that would maintain visible bubbles within the washer. Air passed through the HONO source line from the start of the experiment to avoid sudden changes in humidity. H_2_SO_4_ was pipetted into the gas washer in a 1:1 molarity ratio with NaNO_2_ to begin HONO_(g)_ production. A 0.2 μm PTFE membrane filter was placed in line with the HONO_(g)_ flow to prevent the aerosolized material from reaching the AOT chamber. For lower HONO_(g)_ concentration experiments, a 0.3 lpm dilution flow was passed through the HONO gas washer. Only 0.02–0.04 lpm of this flow was directed toward the chamber, with the remaining 0.26–0.28 lpm routed to exhaust. The resulting HONO_(g)_ concentrations were 3–9 ppm_v_.

It should be noted that the droplet is tweezed within the boundary layer of the AOT chamber ∼100 μm above the glass coverslip and thus likely experiences reduced vapor transport. As such, the HONO_(g)_ concentrations reported here from gas flow dilution calculations should be taken as an upper limit for HONO_(g)_ droplet exposure.

### Data Acquisition and Processing

The NO_3_^–^ peak area, centered at 1035 cm^–1^ in the Raman spectrum, was monitored to track the reactive uptake and oxidation of HONO_(g)_ to HNO_3_ due to the greater mode intensity and sharpness compared to the weaker NO_2_^–^ Raman mode at 1345 cm^–1^. In SO_4_^2–^ and HSO_4_^–^ droplets, the SO_4_^2–^ mode centered at 980 cm^–1^ is close to—but distinct from—the NO_3_^–^ Raman mode. However, HSO_4_^–^ exhibits a broad, low-intensity mode centered at 1051 cm^–1^ that overlaps significantly with the NO_3_^–^ mode. A composite reference spectrum of the major Raman modes used in this work is shown in Figure S2.

Aqueous droplets ranging from 0 to 4 M NaNO_3_ were used as Raman calibration solutions, with the total ionic strength of the calibration droplets remaining at 4 M through the addition of NaCl. The HONO_(g)_ uptake and oxidation rate were calculated by finding the change in NO_3_^–^ concentration versus time from the peak area of the NO_3_^–^ Raman mode in M s^–1^. The uncertainty ranges presented are determined from the standard deviation between replicate experiments.

Raman spectra were collected in LightField software and exported to MATLAB (2022a). Built-in MATLAB functions were used to baseline and denoise spectra. Spectra were normalized to a background peak (987 cm^–1^) from the immersion oil placed on the microscope objective to account for day-to-day changes in the laser power and detector sensitivity for quantitative analysis. Raman spectra shown here are not normalized to retain features of interest. Whispering gallery modes (WGMs) are stimulated Raman modes that resonate with the droplet’s circumference and greatly increase the Raman signal at specific wavelengths. WGMs were removed by identifying rapid changes in peak area when present in a spontaneous Raman mode being used to find a component’s concentration.

Retrieving droplet size and refractive index through whispering gallery mode analysis is discussed in detail for this system by Gorkowski et al.^36,37^ In short, the wavelength position of each WGM is highly sensitive to the droplet size and refractive index. By minimizing the error between calculated and measured WGM positions using a Mie scattering optical model, it is possible to find the droplet size and refractive index with high accuracy. This enables the determination of periods of droplet growth or shrinking, the overall change in chemical composition (from the refractive index), and changes in droplet morphology if these occur due to liquid–liquid phase separation. Not all droplets display WGMs consistently enough for adequate size and refractive index analysis. However, the average radius of droplets trapped ranges from 3 to 4 μm.

### Chemicals and Gases

A 0.3 M aqueous solution of sodium nitrite (Sigma-Aldrich, 99.5%) with 1:1 molar equivalence of sulfuric acid (Supelco, ACS grade) in a gas washer was used to create HONO_(g)_. Sodium nitrate (Sigma-Aldrich, ≥99.0%) was used in droplet and bulk solution calibration of the Raman spectra. Sodium chloride (VWR, ≥99.0%), sodium bisulfate (Sigma-Aldrich, technical grade), ammonium bisulfate (BTC, 99.9%), ammonium sulfate (Sigma-Aldrich, ≥99.0%), and magnesium sulfate (Sigma-Aldrich, ≥99.5%) were used to make aqueous solutions for droplet generation. 18.2 MΩ Milli-Q water was used in all cases. Air flows consisted of ultrazero air (Matheson), prepurified nitrogen (Matheson, 99.998%), ultrapure oxygen (Matheson, 99.994%), or purified house air. Ozone was generated by passing ultrazero air through an HTU-500 ozone generator. A 4 L glass bottle fitted with two inlets was used for an ozone and air dilution mixing chamber before flowing to the aluminum mixing chamber of the AOT via Teflon tubing and stainless steel fittings. Ozone was diluted to 0.5% by volume. Glutaric acid (TCI, ≥99.0%) and 3-methylglutaric acid (TCI, ≥99.0%) were added to select bulk solutions for droplet generation. (−)-α-Pinene (Aldrich, analytical standard) was used as an organic vapor precursor to add secondary organic matter produced by ozonolysis to aqueous tweezed droplets.

## Results

### Inorganic Aqueous Droplets

#### Oxidation of Sodium Nitrite in Aqueous Droplets

Baseline bulk Raman spectra for NaNO_3_ solutions show a strong NO_3_^–^ Raman mode centered at 1035 cm^–1^, while bulk solutions of NaNO_2_ show a shorter and broader NO_2_^–^ mode at 1345 cm^–1^ (Figure S2). In a droplet generated from a solution of NaNO_3_ or NaNO_2_, only the NO_3_^–^ mode is reliably visible. The observation of nitrate but not nitrite in tweezed droplets produced from NaNO_2(aq)_ was thus unexpected and was investigated further.

Aqueous nitrite was observed to oxidize slowly to nitrate within a tweezed aqueous supermicron droplet (Figure S3). This oxidation rate of nitrite by oxygen was calculated across four replicate 4 M sodium nitrite droplets using ultrapure O_2_ as the surrounding medium. Each droplet was trapped individually, and the rate of NO_2_^–^ loss was monitored by subtracting the formed NO_3_^–^ from the initial nitrite concentration (assuming stoichiometric equivalence and no droplet concentration enhancement for NaNO_2_ from the bulk solution). The observed NO_3_^–^ formation rate was (8.0 ± 5) × 10^–4^ M s^–1^. The rate constant determined for this reaction of aqueous nitrite autoxidation was 0.76 ± 0.2 M^–1^ s^–1^, assuming NaNO_2_ is in excess to dissolved O_2_ and using the equation derived by Hunt et al.:^39^


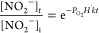
E1where *P*_O_2__ is the partial pressure of O_2_ in atm, *H*′ is the Henry’s law constant adjusted to ionic strength in M atm^–1^, *k* is the rate constant in M s^–1^, and *t* is the time of the reaction in seconds. *H*′ for oxygen was determined by using the Sechenov equation and found to be 9.86 × 10^–4^ M atm^–1^. This kinetic equation was first presented by Smith et al.^40^ and describes the uptake of a gaseous reactant via the loss of a condensed-phase species while accounting for the nonreactive accommodation of the gaseous reactant into the aerosol liquid phase. The reactive–diffusive length of oxygen exceeds the diameter of the droplet, and therefore, the kinetics is volume-limited and not surface-limited. More details on the derivation of this equation can be found in the Supporting Information.

To confirm that O_2_ was the primary oxidant responsible for NO_2_^–^ oxidation or otherwise critical to the reaction mechanism, three 4 M NaNO_2_ droplets were levitated in a N_2_ atmosphere. These droplets exhibited no nitrite oxidation outside a temporary initial period immediately following nebulization that introduced some room air into the chamber (Figure S4). The ability to reliably distinguish between NO_2_^–^ and NO_3_^–^ and quantify their separate concentrations using Raman spectroscopy enabled these direct measurements of the spontaneous oxidation of HONO to HNO_3_ in aerosol droplets.

#### Rate of HONO Uptake to Inorganic Aqueous Droplets

Saturated NaCl droplets (approximately 6 M) with a bulk pH of 6.1 were trapped and allowed to equilibrate to chamber conditions for a period of 5 to 15 min before being exposed to HONO_(g)_. Upon exposure to HONO, rapid formation of NO_3_^–^ was visible in the Raman spectra of the droplets (Figure S5a). The rate of this NO_3_^–^ production was found to be 0.023 ± 0.01 M s^–1^ across four individual droplet experiments under purified house air. No NO_2_^–^ was observed in the droplet Raman spectra. Figure S5b shows the linear regression of the NO_3_^–^ concentration (from the Raman peak area) versus time for the droplet experiment shown in Figure S5a. The rate of NO_3_^–^ formation was monotonic before reaching saturation at approximately 10 M, near the saturation limit of NaNO_3_ in ∼20 °C bulk water.

To determine whether uptake of HONO occurred on acidic droplets more representative of atmospheric particulate matter, 4 M NaHSO_4_ (bulk pH = 0.02) and NH_4_HSO_4_ (bulk pH = 2.38) droplets were used. The rate of NO_3_^–^ production in NH_4_HSO_4_ and NaHSO_4_ droplets was 0.024 ± 0.01 M s^–1^ across three NaHSO_4_ droplet experiments and one NH_4_HSO_4_ droplet experiment. This matches the NO_3_^–^ peak formation rate observed in pH-neutral saturated NaCl droplets. The NH_4_HSO_4_ droplet did not exhibit a different HONO uptake rate (from the NO_3_^–^ production rate) compared to its NaHSO_4_ counterparts. However, NH_4_HSO_4_ droplets are more difficult to trap, and the Raman peak area of the HSO_4_^–^ peak at 1051 cm^–1^ is more variable, likely due to the semivolatile nature of NH_4_^+^/NH_3_. Large changes in droplet composition and therefore water content and size alter how the laser beam interacts with the droplet, destabilizing the optical trap. Thus, the NaHSO_4_ droplet experiments were preferred for determining the NO_3_^–^ formation rate.

**Figure 1:**
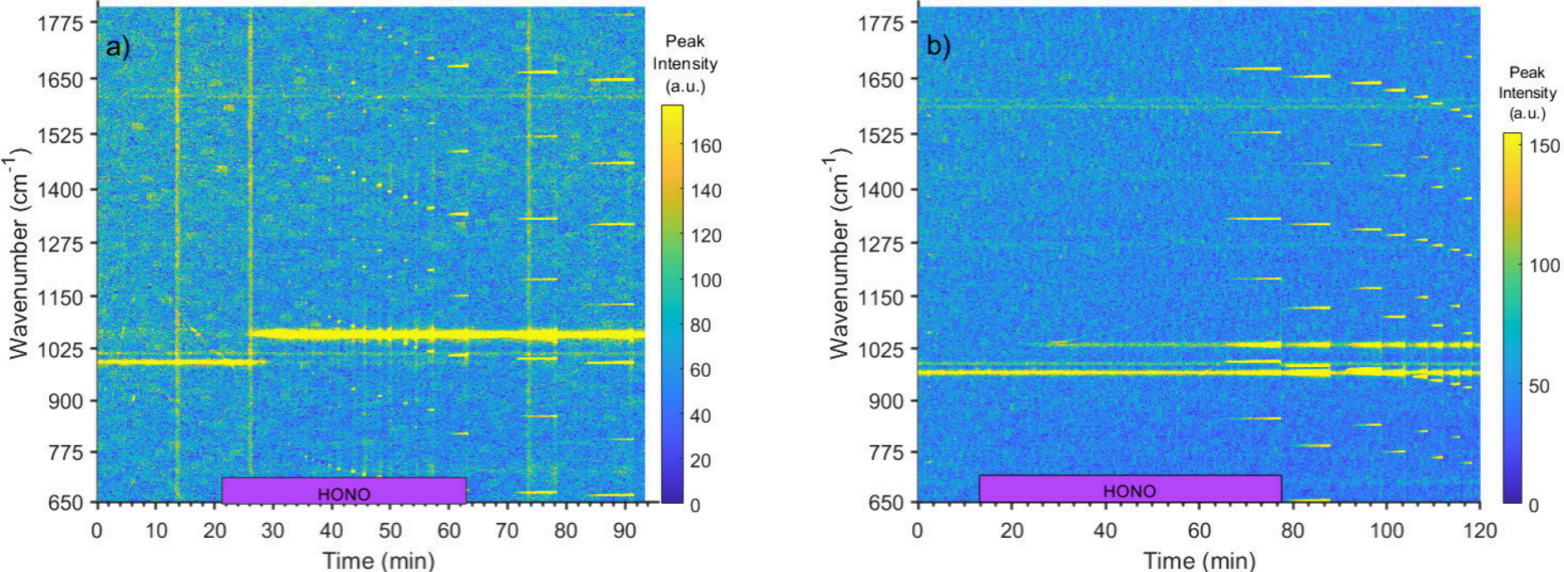
(a) Raman spectral time series for a 4 M NaHSO_4_ droplet exposed to HONO_(g)_. HONO_(g)_ exposure is marked by a purple bar at the bottom. NO_3_^–^ is seen at 1035 cm^–1^. SO_4_^2–^ is visible before and just after HONO_(g)_ exposure at 980 cm^–1^. A faint HSO_4_^–^ peak is visible at around 1051 cm^–1^ before HONO_(g)_ exposure. WGMs are visible as short bright horizontal streaks across the spectral window. (b) Raman data for a 2.5 M MgSO_4_ with 1 M NaCl droplet exposed to HONO_(g)_. SO_4_^2–^ is visible at 980 cm^–1^ and NO_3_^–^ at 1035 cm^–1^. A shallow peak around 987 cm^–1^ is a background peak in both spectral time series, likely from the microscope immersion oil. The large vertical bands of high intensity in (a) are the result of room light interference; they increase the baseline but do not affect peak area calculations.

An experiment using a 4 M NaHSO_4_ droplet is shown in Figure 1a. A SO_4_^2–^ mode is visible at 980 cm^–1^ before HONO_(g)_ exposure, with a smaller HSO_4_^–^ mode at 1051 cm^–1^. After HONO_(g)_ exposure (purple bar), the HSO_4_^–^ mode is quickly replaced by the NO_3_^–^ mode (1035 cm^–1^). The disappearance of the SO_4_^2–^ mode at 980 cm^–1^ is likely the result of the titration of SO_4_^2–^ to HSO_4_^–^ caused by the uptake of HONO_(g)_. Since the HSO_4_^–^ Raman mode overlaps with that of NO_3_^–^, at least some of the NO_3_^–^ signal growth is the result of SO_4_^2–^ protonation. However, it is unlikely that the HSO_4_^–^ Raman signal significantly interferes with the NO_3_^–^ peak formation rate due to its lower Raman scattering efficiency and the fact that the rate of NO_3_^–^ mode increase is statistically identical to the rate of NO_3_^–^ peak formation in NaCl droplets. If the conversion of SO_4_^2–^ to HSO_4_^–^ impacted the NO_3_^–^ peak growth rate, there would be an apparent acceleration in NO_3_^–^ formation compared with NaCl droplets.

Purified house air was used for three of these HSO_4_^–^ droplet experiments (two using NaHSO_4_ and one using NH_4_HSO_4_), while ultrazero air was used for the fourth, a NaHSO_4_ droplet. The NO_3_^–^ mode formation rate under ultrazero air conditions was 0.036 M s^–1^, compared to 0.020 ± 0.009 M s^–1^ for house air, indicating that it was not gaseous trace contaminants leading to HONO_(p)_ (particle phase) oxidation in the droplet.

Since bulk measurements by a pH meter only capture the initial pH of the nebulized solution, the E-AIM aerosol thermodynamic model was used to better understand the final pH of these droplets following HONO_(g)_ uptake using the Raman mode areas to determine product concentrations.^38,41,42^ The pH of the NaCl droplets was determined after HONO_(g)_ uptake to be −0.38. A 4 M NaHSO_4_ droplet was calculated to have a pH of −0.34 postexposure. Thus, the final pH values of these two droplets are expected to be similar.

HONO_(g)_ uptake to droplets composed of MgSO_4_ or (NH_4_)_2_SO_4_ was also studied. Magnesium provides a divalent cation compared to the monovalent sodium cation, allowing us to examine possible surface charge effects on the reactive uptake of HONO. MgSO_4_ droplets also proved to be generally more stable in long-term trapping than their Na_2_SO_4_ counterparts. MgSO_4_ droplets saturated before reaching a concentration of 4 M. As a result, the MgSO_4_ droplets were 2.5 M, with 1 M NaCl added to promote trapping by increasing the ionic strength and thus the hygroscopicity. (NH_4_)_2_SO_4_ is soluble at 4 M, and no NaCl was added. This provided a monovalent cation option that was soluble at the high concentration of sulfate/bisulfate used in the previous experiments, whereas Na_2_SO_4_ was not soluble enough. The pH of the MgSO_4_ solution was 7.74, and that of the (NH_4_)_2_SO_4_ solution was 5.28. The uptake rate for the MgSO_4_ and (NH_4_)_2_SO_4_ droplets was (3.7 ± 1) × 10^–4^ M s^–1^ across the three droplets studied: two MgSO_4_ and one (NH_4_)_2_SO_4_. This rate is close to 2 orders of magnitude lower than the rates measured for NaCl and HSO_4_^–^ droplets.

Figure 1b shows the Raman spectral time series for an example MgSO_4_ droplet. A significant difference between the MgSO_4_ and NaHSO_4_ experiments is that a SO_4_^2–^ Raman mode (980 cm^–1^) remained throughout the entire HONO_(g)_ exposure in SO_4_^2–^ droplets. The SO_4_^2–^ mode area did decrease over the experimental period. This contrasts with the HSO_4_^–^ droplets, where the SO_4_^2–^ mode rapidly returned to the baseline. This difference in SO_4_^2–^ mode behavior is likely explained by comparatively more SO_4_^2–^ being present in MgSO_4_ versus the NaHSO_4_ droplets and by the fact that the NO_3_^–^ formation rate is much lower in the MgSO_4_ droplets. This is likely because the higher concentration of SO_4_^2–^ titrates the produced acid and buffers the pH. Unlike the faster-reacting systems, the NO_3_^–^ mode increase and SO_4_^2–^ mode decrease never ceased during droplet exposure to HONO_(g)_ for MgSO_4_ droplets.

For the MgSO_4_ experiments shown in Figure 1b, most WGM activity follows the cessation of the flow of HONO_(g)_ into the chamber, limiting the analysis of the size and refractive index changes during the reaction. However, a brief period of WGM activity about 1 min after droplet capture allowed the initial droplet parameters to be determined. Analysis of WGMs for this MgSO_4_ droplet (Figure S6b) showed an initial droplet radius of 3780 nm. Following HONO uptake, the droplet decreased to 3520 nm, followed by slow growth back to 3760 nm after the cessation of HONO_(g)_. The refractive index showed an increase from the initial 1.365 to 1.375 during HONO_(g)_ exposure, then a decrease from 1.375 to 1.370 after the flow of HONO_(g)_ stopped. The changes after HONO(g) exposure could be the result of increased droplet hygroscopicity, as water uptake would decrease the refractive index toward the value of 1.33 for pure water.

A 4 M H_3_PO_4_ droplet (bulk pH 0.10) was used as an exclusively weak acid–base species and a triprotic acid comparison. The NO_3_^–^ formation rate was measured to be 0.039 M s^–1^. This is near the higher end of the rates observed in the NaHSO_4_ droplets.

Several NaHSO_4_ droplets were exposed to HONO_(g)_ after the AOT chamber was purged of O_2_ by high-purity N_2_ gas flow. No apparent uptake of HONO_(p)_ was detected in either the formation of a NO_3_^–^ or NO_2_^–^ Raman mode or the titration of SO_4_^2–^ to HSO_4_^–^ that would indicate the production of a strong acid. Figure S7 shows an example NaHSO_4_ droplet under a N_2_ atmosphere with HONO_(g)_ exposure.

**Figure 2:**
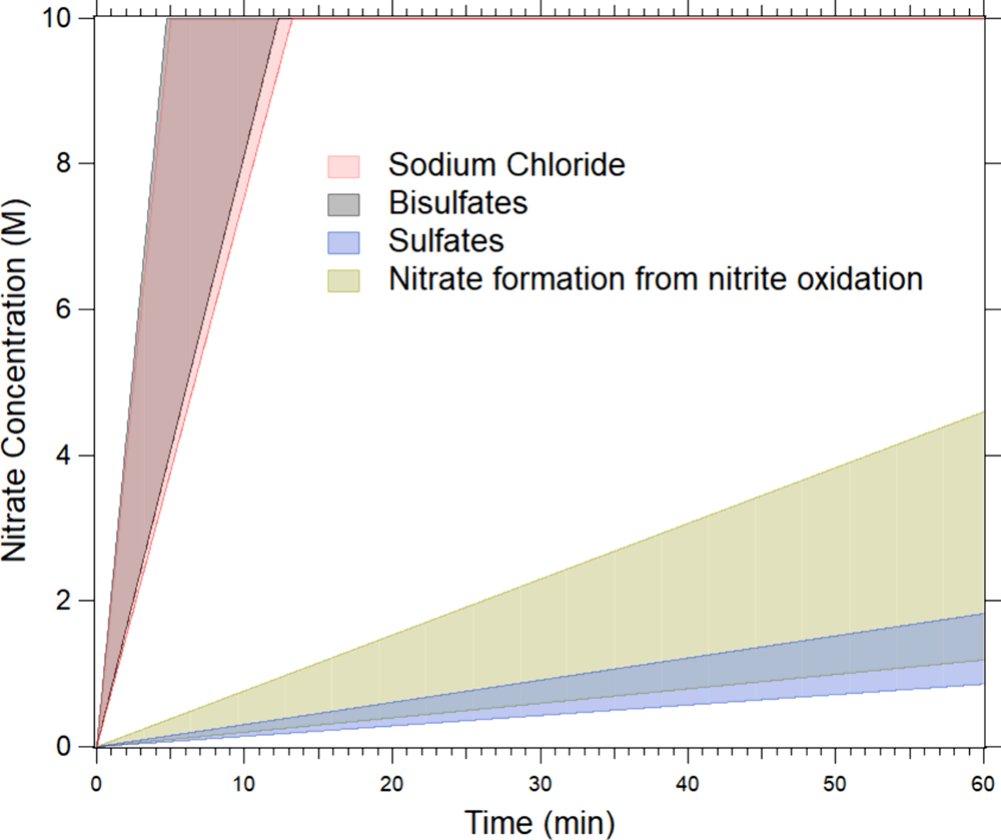
Increase in NO_3_^–^ concentration versus HONO exposure time to compare the nitrate formation rates during HONO uptake for three inorganic droplet systems shown by red (NaCl), gray (HSO_4_^–^), and blue (SO_4_^2–^) shaded regions. Gold represents NO_3_^–^ formation in a 4 M NaNO_2_ droplet exposed to air containing O_2_. Shaded regions represent the standard deviation determined for three to four replicate droplets. The *y*-axis maximum is set to 10 M, near the NaNO_3_ water solubility limit and the highest nitrate concentration measured.

The observed rates of nitrate production from HONO_(g)_ uptake from the three primary inorganic droplet systems studied (NaCl, NaHSO_4_ and NH_4_HSO_4_, and MgSO_4_ and (NH_4_)_2_SO_4_) are compared in Figure 2. Also included is the rate of the formation of the NO_3_^–^ peak from 4 M NaNO_2(aq)_ droplets under the O_2(g)_ conditions. The shaded regions represent the standard deviation of the NO_3_^–^ formation rates for each system from replicate experiments. The NO_3_^–^ formation rates in NaCl and HSO_4_^–^ droplets are identical within experimental uncertainty, while the rate in SO_4_^2–^ droplets overlaps substantially with that observed for O_2_ oxidation of NaNO_2_.

#### Effect of Reduced HONO Concentration

To better understand the dependence of the NO_3_^–^ formation kinetics on the concentration of HONO_(g)_, a diluted flow of HONO_(g)_ was introduced into the chamber. The resulting HONO_(g)_ concentrations were approximately 5 to 15 times lower (3–9 ± 3 ppm_v_) than in the preceding experiments (46 ± 13 ppm_v_). The aqueous inorganic seed droplets tested were composed of NaCl, NaHSO_4_, or MgSO_4_. The NO_3_^–^ formation rate observed on the saturated NaCl droplet was 9.7 × 10^–5^ M s^–1^ at 3 ppm_v_ HONO_(g)_, compared to 2.3 × 10^–2^ M s^–1^ at 46 ppm_**v**_ HONO_(g)_. The 4 M NaHSO_4_ droplet exhibited a NO_3_^–^ formation rate of 4.0 × 10^–4^ M s^–1^ at 9 ppm_v_ HONO_(g)_ versus a NO_3_^–^ formation rate of 2.4 × 10^–2^ M s^–1^ at 46 ppm_**v**_ HONO_(g)_. Lastly, the 2.5 M MgSO_4_ droplet experienced a NO_3_^–^ formation rate of 6.7 × 10^–5^ M s^–1^ at 6 ppm_v_ HONO_(g)_ compared to 3.7 × 10^–4^ M s^–1^ at the higher concentration of 46 ppm_v_. These represent approximate reductions in observed HONO → NO_3_^–^ oxidation rates by factors of 227, 61, and 5.5, respectively.

#### Considering Literature Reaction Mechanisms for Aqueous Nitrous Acid Oxidation

The reaction of nitrite with oxygen had been studied in aqueous bulk solution but not reported in aerosol droplets to our knowledge.^34,35,43,44^ Damschen and Martin proposed a rate law that is second order in [HONO] by monitoring oxygen loss within a solution containing millimolar nitrite concentrations at varying pH values:^43^
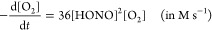
E2From this determined rate law, Damschen and Martin proposed the following reaction mechanism for the oxidation of HONO that first requires HONO dimer formation:^43^
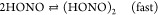
R1
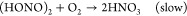
R2These authors found that there was a strong pH dependence for the reaction kinetics, with the reaction accelerating at low pH, and they proposed that it was nitrous acid itself reacting with oxygen and not nitrite. They concluded that due to the second-order rate dependence on HONO, the reaction would be too slow to be relevant in the atmosphere.^43^ Dissolved oxygen could also be rate-limiting if the reaction occurs in the bulk, as was studied in their experiments.

Mudgal et al. summarized two potential reaction schemes for the autoxidation of nitrous acid in aqueous solution by monitoring the loss of oxygen in solution with and without freezing, a process known to accelerate certain reactions.^44^ The first scheme involves complexation of nitrous acid with oxygen on the nitrogen atom in a reversible process, followed by the formation of an intermediate through the addition of another nitrous acid. This intermediate then breaks apart into two nitrate ions and associated protons. Their proposed reaction scheme is as follows:^44^
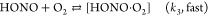
R3
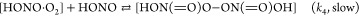
R4
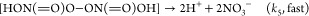
R5The associated rate law for this reaction scheme was determined as follows when [N^III^] ≫ [O_2_] and the pH is fixed:
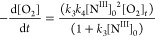
E3where [N^III^] signifies the combined concentration of HONO and NO_2_^–^.^44^ This equation simplifies when 1 ≫ *k*_3_[N^III^]_0_, as indicated by their reaction results showing a clear second-order dependence on [N^III^]_0_, resulting in a rate law that mirrors that proposed by Damschen and Martin,^43^ despite the very different reaction mechanism:
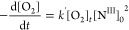
E4

The second scheme Mudgal et al. discussed is the complexation of two nitrous acid molecules via hydrogen bonding in a *cis* configuration that exposes open orbitals on the matching nitrogen atoms with which triplet-state molecular oxygen can form covalent bonds.^44^ This mechanism would result in the following rate law including the dependence on pH and the acid dissociation constant of HONO (*K*_a_):^44^
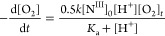
E5This rate law matches that proposed by Takenaka et al., who also studied the rate of nitrous acid oxidation in frozen aqueous solutions (note the first-order dependence on [N^III^]_0_).^34,35^ Ultimately, Mudgal et al. were unable to determine which mechanism is most plausible: whether nitrous acid first bonds with oxygen or another nitrous acid molecule.^44^ They postulated that their first mechanism is more likely under ambient conditions where oxygen concentrations exceed nitrous acid concentrations, whereas the second mechanism is more feasible in the frozen solution experiments conducted by Takenaka et al.^34,35,44^

The bulk solution concentrations of HONO in the previously discussed studies were in the millimolar range, where dissolved O_2_ was generally the limiting reactant. In the case of aerosol droplets with large surface area to volume ratios and exposed to low parts per million to parts per billion gas-phase concentrations of HONO, oxygen is unlikely to be a limiting factor, particularly if the reaction proceeds at the surface. Here, the high collision rate of gas-phase oxygen would far exceed that of HONO under ambient conditions.

A study by Braida and Ong was the first to analyze the dependence of nitrite oxidation/degradation on pH and oxygen prevalence dependence using a bulk bubbler approach.^45^ They determined pseudo-first-order rate constants ranging from 1.2 × 10^–6^ to 1.12 × 10^–4^ s^–1^, with the higher rate constant observed at lower pH and higher oxygen flow rates. Their proposed oxidation pathway^45^ relies on that put forward by Abel et al.:^46^
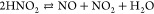
R6
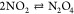
R7
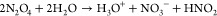
R8Braida and Ong hypothesized a competition between decomposition and oxidation of HNO_2_, where the decomposition products NO and NO_2_ can evaporate prior to oxidation, leading to a discrepancy in their mass balance.^45^ Certain conditions, such as lower oxygen concentrations, can favor decomposition over the oxidation of HNO_2_.

Other literature-reported mechanisms apply first-order kinetics with respect to NO_2_^–^ as the reactant, leading to the following rate laws for NO_2_^–^ → NO_3_^–^ oxidation:^39,47^
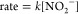
E6
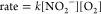
E7The primary goal of these studies was not to determine the mechanism of oxidation of NO_2_^–^. However, they do support the interesting disconnect between varying extents of the decrease in the rate of NO_3_^–^ formation that was apparent in our experiments under reduced HONO_(g)_ concentrations.

#### Droplet-Accelerated Chemistry

Before further kinetic analysis of our experiments, we review the current understanding of droplet-accelerated chemistry that leads to an increase in chemical reaction rates compared to bulk solution. This has been attributed to the unique chemical properties at the air–water interface.^48−52^ Reactants are less solvated at the interface, which can greatly reduce reaction energy barriers.^50^ A local concentration of reactants at the interface is possible, especially for surface-active or less soluble compounds.^53^ This effect of chemical confinement such as at interfaces can greatly accelerate the rates of chemical reactions by over 10^6^ for some systems and even alter the reaction mechanism and thus the rate constant. Wilson and Prophet recently reviewed these effects of microconfinement relevant to aerosol particles and similar systems with roughly micron length scales.^51^ They used the oxidation of NO_2_^–^ by gas-phase O_3_ in an optically tweezed droplet reported by Hunt et al.^39^ to illustrate the modeling of microconfinement-accelerated kinetics to determine the contributions from surface versus bulk reactions. They concluded for this system that bulk reactions cannot explain the observed oxidation rate of NO_2_^–^ to NO_3_^–^ due to O_3_ diffusional limitations, implying an important role of reactions in the surface layer in addition to bulk oxidation.

The pH of the interface is also thought to be very different from that of the bulk; this is an ongoing research question in physical chemistry.^54−56^ The large electric field that manifests at the interface may alter the reaction mechanism and energy barrier height. This electric field has also been proposed to be the cause of spontaneously produced reactants in microdroplets such as ·OH, H_2_O_2_, and aqueous electrons reported in a growing number of recent studies.^57−60^ This notion remains controversial, however.^51^ Note that we ruled out contributions from spontaneously generated oxidants here by using a ·OH scavenger. As gas-phase reactants must be first uptaken onto the air–water interface and then diffuse through this region into the bulk, gas–particle reactions are especially susceptible to these effects of droplet-accelerated chemistry.^51^ As smaller aerosol particles have greater surface area to volume ratios, which would increase any interfacial effects on droplet-accelerated chemistry,^51^ the kinetics of HONO oxidation to HNO_3_ reported here on supermicron droplets likely represents a lower limit to the rate of this reaction on submicron accumulation-mode aerosol. Our type of AOT can only trap supermicron-diameter droplets, and the real-time Raman analysis capabilities enabled our observation of the prompt conversion of HONO to HNO_3_ in the form of NO_3_^–^.

#### Oxidation of HONO to HNO_3_ at the Air–Droplet Interface

The rate of HONO_(g)_ uptake on a droplet, as measured by the NO_3_^–^ formation rate, is not limited by the p*K*_a_ of HONO that could inhibit aqueous uptake in these experiments. The observed pH dependence is the reverse of what would be expected if considering just the pH and p*K*_a_ of HONO and droplet systems, where HONO partitions into the droplet only after deprotonation. Table 1 provides a summary of the reaction conditions and observed nitrate formation rates for the inorganic salt droplet experiments. It is clear from these data that acidic droplets accelerate NO_3_^–^ formation from HONO uptake. The rate of NO_3_^–^ formation from NaNO_2(aq)_ oxidation by O_2_ is similar to the NO_3_^–^ formation rate in SO_4_^2–^ droplets but 2 orders of magnitude lower than those in the more acidic/unbuffered HSO_4_^–^ and NaCl_(aq)_ droplets, where HNO_3_ formation from HONO_(g)_ uptake acidifies the NaCl droplets. The presence of a prevalent buffer species, in this case SO_4_^2–^, is enough to negate the acid-accelerated oxidation of HONO_(p)_ in the droplet by titrating the HNO_3_ produced from HONO oxidation.

**Table 1:** Summary of Aqueous Inorganic Droplet Composition and Kinetics with High HONO Exposure (46 ± 13 ppm_v_)

droplet composition	conc. 1 (M)	conc. 2 (M)	no. of droplets	initial pH (bulk)	reactive uptake coefficient	NO_3_^–^ formation rate (M s^–1^)
NaCl	saturated (∼6)	–	4	6.10	(1.4 ± 0.6) × 10^–4^	0.023 ± 0.01
bisulfates (Na^+^, NH_4_^+^)	4	–	4	–0.02	(1.4 ± 0.6) × 10^–4^	0.024 ± 0.01
(NH_4_)_2_SO_4_	4	–	1	5.28	(2.3 ± 0.6) × 10^–6^	(3.7 ± 1) × 10^–4^ (with MgSO_4_)
MgSO_4_ and NaCl	2.5	1	2	7.74	(2.3 ± 0.6) × 10^–6^	(3.7 ± 1) × 10^–4^ (with (NH_4_)_2_SO_4_)
H_3_PO_4_	1	–	1	0.10	(2.4 ± 0.6) × 10^–4^	0.039

The question is why HONO_(g)_ partitions to acidic droplets at all when the pH is below the p*K*_a_ of HONO. HONO is expected to remain in the gas phase in this scenario. This is why HONO uptake to atmospheric aerosol particles has been assumed to be negligible, except for large dilute and less acidic cloud droplets. Considering that NO_3_^–^ formation is what was directly measured here and NO_2_^–^ formation was not measured during HONO_(g)_ exposure, we are proposing that HONO_(ads)_ (adsorbed at the interface) is oxidized to HNO_3_ at the interface of an acidic droplet where elevated local adsorbed concentrations of HONO and O_2_ promote oxidation to NO_3_^–^. This oxidation would negate the evaporation of the semivolatile HONO when NO_2_^–^ is protonated given the much lower p*K*_a_ and higher Henry’s law constant of HNO_3_.

For SO_4_^2–^ to act as a hindrance to accelerated interfacial oxidation of HONO_(ads)_ to HNO_3_, the oxidation would have to be slower than the deprotonation of HONO_(ads)_ by SO_4_^2–^ but competitive with or faster than the desorption of HONO_(ads)_ on the droplet interface. As solutes are less solvated in the interfacial region versus the bulk, this would likely reduce the acid dissociation of HONO.

A mathematical estimation of how well a second-order HONO_(g)_ dependence describes the observed kinetics can be achieved by taking the ratio of the observed rate from the high and low HONO_(g)_ reactions using the rate law from eq E4, as shown in eq E8:
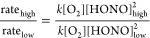
E8This involves some assumptions, most notably that the concentration of O_2(p)_ is equivalent for all HONO uptake experiments and that the HONO_(p)_ concentration increases proportionally to HONO_(g)_ exposure.

An equivalent approach is used for eq E7 as the ratio of the high-to-low concentration experiments for testing the rate of NO_2_^–^ oxidation for a first-order dependence on HONO/NO_2_^–^. In this approach, any resulting discrepancy between the left- and right-hand sides of eq E8 can be quantified. Any observed rate at high HONO concentration that is greater than any change in reactant concentration and an assumed reaction order would predict indicates the potential for surface acceleration to affect the rate constant or a restriction of reactants to the surface. For saturated NaCl droplets, there is a discrepancy of 15 ± 7 for a first-order HONO_(g)_ dependence or 1.0 ± 0.4 using a second-order dependence on HONO_(g)_ (uncertainty propagated from the standard deviation of the measured NO_3_^–^ formation rate). If the reaction is first-order with respect to [HONO], the discrepancy in expected rate between the high- and low-concentration experiments is likely the result of one of two factors: an interfacial concentration of reactants or an increase in the rate constant. The excellent agreement obtained with the second-order in [HONO] rate law indicates that the change in reaction rate versus HONO concentration can be described entirely by the changes in HONO concentration and suggests that the assumption that droplet concentrations of HONO_(p)_ increase proportionally to gas concentrations is valid in this scenario. In either case, it is unlikely that increasing H_3_O^+^ concentrations, as in eq E5, had any major impact on reaction rates given that the 4 M NaHSO_4_ droplet rates varied from their expected ratio by 12 ± 5 and 2.4 ± 1.1 for first- and second-order dependence, respectively. The similarity in these reaction rate variations to the NaCl droplets, particularly for first-order [HONO] dependence as in eq E5, in a droplet with high initial H_3_O^+^ concentrations already present suggests that increased interfacial concentrations or an increase in the rate constant (unrelated to a catalytic effect by H_3_O^+^) is the cause for any potential surface acceleration or concentration enhancement.

For MgSO_4_ droplets, with a 5.5 factor decrease in NO_3_^–^ formation rate resulting from a 7.6 factor decrease in HONO_(g)_, there is no second-order dependence, and no apparent surface acceleration is observed. This suggests that the buffered droplets experience only a reaction mechanism that is first-order in NO_2_^–^ with no potential interfacial enhancement, possibly due to migration of NO_2_^–^ into the bulk volume. A summary of the ratio of reaction rates as specified by eq E8, the first-order in [HONO] equivalent of eq E8, and the HONO concentration ratios used is shown in Table 2.

**Table 2:** Summary of Observed Rate Ratios for High/Low HONO Concentration Kinetic Experiments

		NO_3_^–^ formation rate (M s^–1^)		HONO rate ratio discrepancy	
droplet composition	HONO_(g)_ ratio	high HONO_(g)_	low HONO_(g)_	first-order	second-order
NaCl	15.3	2.3 × 10^–2^	9.7 × 10^–5^	15 ± 7	1.0 ± 0.4
NaHSO_4_	5.1	2.4 × 10^–2^	4.0 × 10^–4^	12 ± 5	2.4 ± 1.1
MgSO_4_ and NaCl	7.6	3.7 × 10^–4^	6.7 × 10^–5^	1.4 ± 0.5	N/A

The reactive uptake coefficient—a measure of the fraction of gas–particle collisions that result in a reaction—for HONO uptake was determined using the method outlined by Smith et al.^40^ (the full equation is given in the Supporting Information). The values show a similar difference between the accelerated and nonaccelerated reaction pathways. The accelerated pathway on acidic/unbuffered droplets results in a reactive uptake coefficient of (1.4 ± 0.6) × 10^–4^ compared to (2.3 ± 0.6) × 10^–6^ for the unaccelerated pathway on neutral/buffered droplets (uncertainty propagated from the standard deviation of measured NO_3_^–^ formation).

These kinetics results have two possible explanations. The first is that HONO/NO_2_^–^ is oxidized following a first-order mechanism, where restriction of HONO to the interface of the droplet results in a surface reaction rate enhancement by a factor of 12–15, a factor that is negated through the deprotonation of HONO and dilution in the bulk volume. Alternatively, the restriction of HONO to the interface or under highly acidic conditions could result in a change in reaction mechanism for the formation of NO_3_^–^ from one that is first-order in [NO_2_^–^] to one that is second-order in [HONO].

An interfacial reaction that follows a [HONO]^2^ rate law could resolve the long-standing confusion around the exact mechanism of nitrite oxidation in bulk aqueous solution reported in the literature that was discussed in the previous section, as there are two competing reaction pathways possible under different reaction conditions. The faster pathway results from a HONO-based complex (eqs E2–E4 and R1–R5) and the slower pathway from a direct reaction of NO_2_^–^_(aq)_ with O_2(aq)_ (eqs E5–E7) or other first-order in [NO_2_^–^] mechanism. In aerosol, the pH of the droplet can determine the prevailing reaction pathway by restricting the reaction to the interface or having it proceed in the bulk volume by dissolved constituents. If HONO remains protonated, this should promote the interfacial reaction pathway, providing an explanation for why the formation of HNO_3_ is faster in unbuffered droplets that become acidified by HONO uptake and autoxidation to HNO_3_.

The probable second-order [HONO] dependence of the faster interfacial pathway does suggest that there are limits to its implications for atmospheric chemistry and the fate of HONO. The experimental setup used here is incapable of creating HONO at low enough concentrations corresponding to the polluted boundary layer for sufficient time periods that allow HNO_3_ formation to be observed and the kinetics and HONO dependence to be determined. However, the evidence that this reaction proceeds directly at the interface suggests that the atmospheric HONO_(g)_ concentration required for this autoxidation to proceed with an appreciable rate may be much less than concluded in the previous literature. The previously proposed rate limitation caused by available dissolved O_2_ would also not be a factor for this interfacial reaction of HONO + O_2_.

#### Evidence of HONO Complex Formation in Literature

Prior studies can provide insights into the most likely mechanism behind the observed autoxidation of nitrous acid. Bunton et al. explored the isotopic exchange of oxygen atoms between water and nitrous acid, absent oxygen, in the 1950s.^61^ In that work, they proposed the following mechanism under acidic conditions:
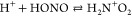
R9
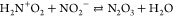
R10The equilibrium would result in the exchange of isotopically labeled oxygen with nitrous acid. The goal of their work was not to explore the next step toward oxidation, but they did produce a rate law for the above mechanism:
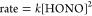
E9This rate law closely matches those proposed by Damschen and Martin^43^ and Mudgal et al.^44^ and supports the conclusion that a spontaneous reaction between two nitrous acid molecules is feasible under the right conditions. The elevated concentration of HONO at the droplet–air interface would promote this self-reaction, but this had not been previously considered by the prior experiments in bulk solution. It is unclear, however, whether the reactive species would be the protonated HONO ion (H_2_N^+^O_2_) or N_2_O_3_ in the above reaction or possibly the HONO dimer in eq R2. Oxidation via N_2_O_3_ would result in the following reaction as proposed by Morrison et al.:^62^
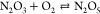
R11This reaction would then facilitate the formation of HNO_3_/NO_3_^–^ through the exothermic hydrolysis of N_2_O_5_.^63−65^ The possible role these intermediates may play in the rapid conversion of HONO to HNO_3_ via O_2_ is unexplored by any literature of which we are aware. The spontaneous oxidation of HONO to NO_3_^–^ was observed only with O_2_ present, implying that O_2_ is required. It should also be reiterated that the goal of these latter two studies by Bunton et al.^61^ and Morrison et al.^62^ was not explicitly to monitor the autoxidation of HONO, and any insights from our AOT experiments and the underlying mechanism of HONO oxidation should be taken with some caution. However, these mechanisms would explain the acid-accelerated kinetics and the second-order dependence on [HONO] observed here and in other studies and support that the reaction largely involves protonated or complexed HONO at the interface instead of NO_2_^–^ in the bulk.

The species H_2_N^+^O_2_ in eq R9 has been observed in other studies and can be used in synthesis applications for the nitration of organics.^66−71^ The p*K*_a_ of H_2_N^+^O_2_ is estimated to be −10,^66^ but it is present in solutions with pH < 2.2.^68^ This pH range is applicable to the bisulfate droplets and likely the sodium chloride droplets as well following sufficient HONO uptake and HNO_3_ formation. Given the significant SO_4_^2–^ signal observed in the buffered sulfate droplets even after HONO exposure and the lack of a HSO_4_^–^ mode, it is unlikely that these droplets reach such a low pH. H_2_N^+^O_2_ may form at the interface due to its very different chemical environment, although the cation would be expected to diffuse into the bulk and perhaps dissociate.

The HONO experiments presented in our study are the first to include high concentrations of dissolved solutes beyond HONO or H_2_O. This provides a unique observation regarding SO_4_^2–^ and HONO. NO_2_^–^ is the stronger conjugate base and should retain the proton in solution with SO_4_^2–^. However, the SO_4_^2–^ Raman peak (980 cm^–1^) disappears during HONO exposure in the case of HSO_4_^–^ droplets or decreases in SO_4_^2–^ droplets. This permanent reduction in the SO_4_^2–^ signal must be from the formation of HNO_3_. As such, the titration of SO_4_^2–^ to HSO_4_^–^ could be assumed to happen via protonation from the HNO_3_ produced by HONO autoxidation. SO_4_^2–^ interrupting this process to slow the acid-catalyzed oxidation of HONO could provide evidence that H_2_N^+^O_2_ is the major reaction intermediate, as it is a strong enough acid to protonate SO_4_^2–^. If H_2_N^+^O_2_ is not the intermediate, then it is feasible that rapid equilibrium exchanges of the proton between the intermediate HONO complex species (HONO)_2_ (eq R2), [HONO·O_2_] (eq R4), and H_2_N^+^O_2_ (eq R7) and SO_4_^2–^ could be enough to destabilize the complexes and reduce their ability to react with O_2_ or promote the movement of HONO from the surface layer into the bulk, where reactant concentrations are reduced; the kinetic regime shifts to first order with respect to the nitrogen species.

### Effect of Addition of Oxidized Organics to Droplet

#### HONO Uptake in the Presence of Organic Aerosol Matter

A surface reaction may be affected by a change in interfacial composition, whether chemically via the presence of surface-active organic components in the aqueous medium or through a physical morphology or phase change. To better determine the limits of the surface conversion of HONO to HNO_3_ by O_2_ in the presence of an organic surface component, droplets containing various proxies for atmospheric organic aerosol were studied, including glutaric acid, 3-methylglutaric acid (3MGA), and secondary organic aerosol (SOA) from α-pinene vapor oxidized by O_3_. If a liquid–liquid phase separation occurs, the organic components would form a shell phase surrounding the aqueous phase, as we have observed in our prior AOT experiments from analysis of the WGMs (a.k.a. morphology-dependent resonances).^36,37^ Acidification is known to drive such a morphology change for glutaric acid and 3MGA, which are weak acids with p*K*_a_ values of 4.34 and 4.27, respectively.^72,73^

A droplet containing 3 M (NH_4_)_2_SO_4_ and 1.5 M 3MGA was used to determine the HONO uptake/oxidation rate in the presence of a weak organic acid and whether a liquid–liquid phase separation is caused by exposure to HONO_(g)_.^36,37,74^ A NO_3_^–^ formation rate of 0.015 M s^–1^ was observed, within the range observed for the HSO_4_^–^ and NaCl droplets under similar conditions (Figure S8). Similarly, a 2 M (NH_4_)_2_SO_4_ and 2 M glutaric acid droplet with an initial pH of 2.56 had a NO_3_^–^ formation rate of 0.024 M s^–1^. These organic acid droplet experiments demonstrate that when SO_4_^2–^ is paired with an organic acid, the rate of uptake of HONO_(g)_ is the same as seen on acidic inorganic droplets. The presence of additional labile protons from the weak organic acids may be enough to offset the hindrance from a buffer, such as SO_4_^2–^ on acid-accelerated NO_3_^–^ formation. Droplet morphology changes during HONO_(g)_ exposure are not clear-cut. However, visible changes in the WGMs can be seen with exposure to HONO_(g)_ compared to baseline conditions, suggesting a possible morphology shift to a core–shell droplet with the organic phase forming the shell around the aqueous inorganic core.

To simulate more realistic atmospheric aerosols that contain complex mixtures of organic compounds from terpene oxidation, two inorganic aqueous seed droplets—one saturated NaCl and one 4 M NaHSO_4_—were exposed to a period of α-pinene ozonolysis followed by HONO_(g)_ exposure. The α-pinene liquid (50 μL) was placed directly into the AOT upper-chamber reservoir, and the flow of O_3_ was brought through the conditioned air lines, bypassing the HONO source. The ozonolysis of α-pinene continued until a morphology change was detected through changes in the WGMs. Approximately 30 min (∼20 chamber residence times) was allowed to elapse between the cessation of O_3_ flow and the initialization of the HONO_(g)_ flow.

**Figure 3:**
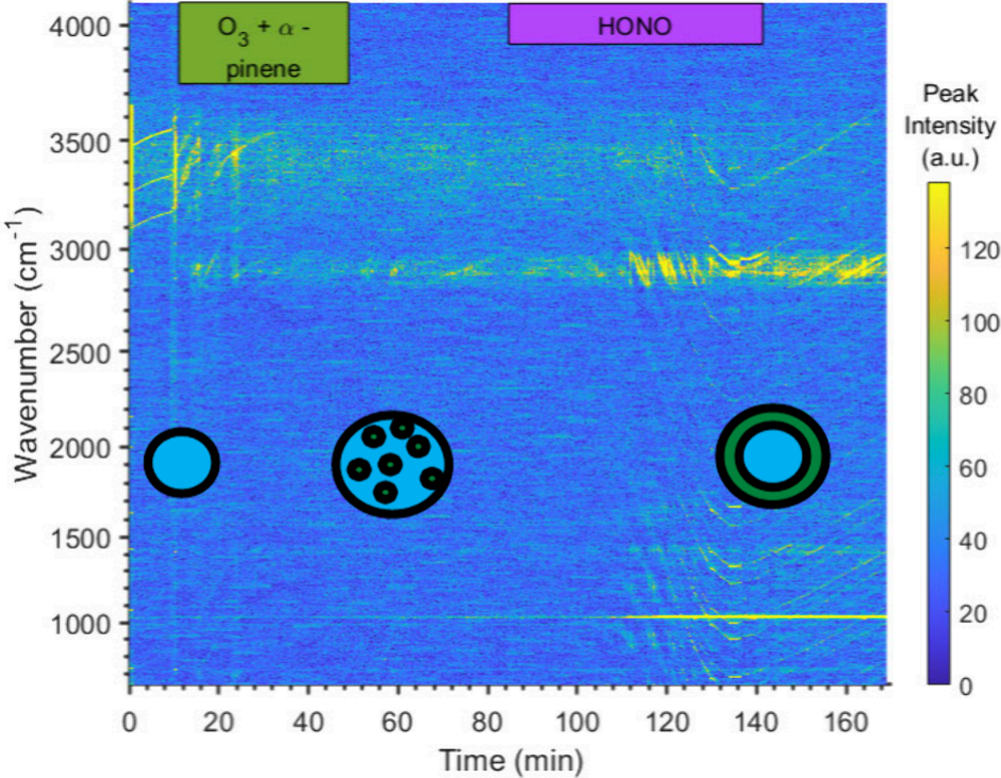
Raman data for a saturated NaCl droplet exposed to two periods of oxidation: α-pinene ozonolysis (green bar) and then HONO_(g)_ exposure (purple bar). NO_3_^–^ mode formation is visible at 1035 cm^–1^. The droplet goes from homogeneous to probable emulsion and to probable core–shell morphology through the experiment. Each morphology is indicated by a diagram.

Figure 3 shows the Raman spectral time series for the saturated NaCl droplet exposed to α-pinene ozonolysis followed by HONO_(g)_ exposure. This droplet undergoes a morphology change during α-pinene ozonolysis as the initial WGMs in the O–H stretch (3100–3600 cm^–1^) of the droplet cease and are replaced by flickering WGMs commonly associated with an emulsion morphology produced when secondary organic aerosol is added to the droplet from terpene oxidation.^37^ Upon HONO_(g)_ exposure, the WGMs return, and there is a marked increase in organic signatures (C–H stretch: 2900 cm^–1^), dampening of the O–H stretch, and increased organic associated modes in the fingerprint region (700–1600 cm^–1^) in addition to strong NO_3_^–^ mode formation (1035 cm^–1^). The WGM fitting remains inconclusive due to poor WGM identification by our analysis algorithm, but this described droplet behavior is strongly associated with the formation of a core–shell morphology, where the organic phase forms a shell around the aqueous core.

This morphological change would lead to two conclusions. The first is that HONO_(g)_ exposure can result in a morphology change in a mixed organic–inorganic aqueous droplet during the production of HNO_3_. The second is that this HNO_3_ production from HONO_(g)_ uptake does not appear to be greatly hindered by either an emulsion of SOA or a core–shell morphology. The NO_3_^–^ formation rate is 0.028 M s^–1^, which is in line with the NO_3_^–^ formation rate in NaCl droplets without SOA.

**Figure 4:**
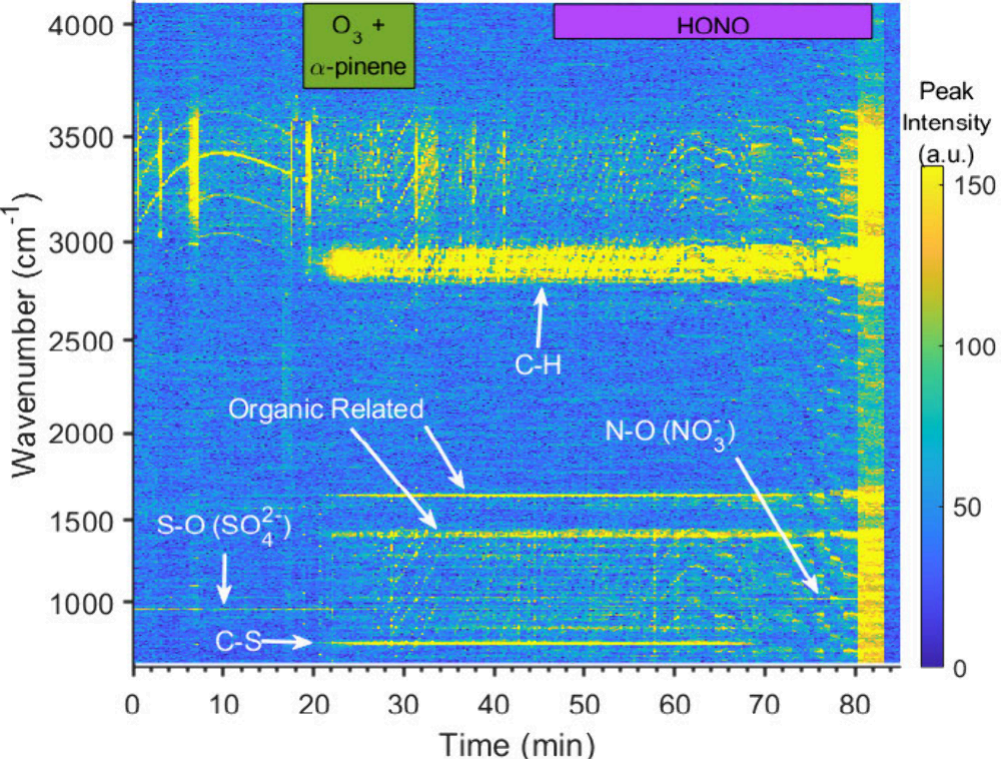
Raman data for a 4 M NaHSO_4_ droplet exposed to SOA produced from α-pinene ozonolysis (green bar) followed by HONO(g) exposure (purple bar). The droplet is lost at 83 min. A strong C–H mode is present at 2900 cm^–1^. The SO_4_^2–^ peak is visible at 980 cm^–1^ and disappears after the first period of α-pinene oxidation. The NO_3_^–^ peak becomes faintly visible at 1035 cm^–1^ after minute 72. Loss of a probable C–S peak at 714 cm^–1^ happens at around 68 min, just before NO_3_^–^ formation is observed.

The Raman study of the 4 M NaHSO_4_ seed droplet with α-pinene ozonolysis SOA (Figure 4) was conducted using similar experimental conditions as for the NaCl droplet described above (Figure 3). This NaHSO_4_ droplet experienced a NO_3_^–^ formation rate of 0.0057 M s^–1^. This rate is intermediate between the two ranges for an inorganic aqueous droplet, acidified or buffered, shown in Figure 2. This is possibly the result of a morphology change in the droplet, as it goes from homogeneous to partially engulfed to core–shell as SOA is continually added during α-pinene SOA uptake. This differs sharply from the saturated NaCl with a α-pinene ozonolysis SOA droplet that did not achieve a core–shell morphology until after HONO_(g)_ exposure was started.

Retrieving the size and refractive index with a Mie model fit of the WGMs for the droplet in Figure 4 was not successful. However, clear changes in WGM behavior are exhibited. Raman spectral changes of note are the fast formation of the C–H mode (centered at 2900 cm^–1^) during α-pinene oxidation, indicating an organic component, and the loss of the SO_4_^2–^ mode (980 cm^–1^) shortly after α-pinene oxidation at 22 min. The acidic nature of this seed droplet accelerated the formation of condensed organic matter compared with the previous NaCl seed droplet. This, along with the immediate core–shell morphology, greatly enhances the C–H and other organic Raman signals. The formation of a NO_3_^–^ mode (1035 cm^–1^) from HONO_(g)_ uptake is at 72 min.

Interestingly, there is a loss of the Raman mode at 714 cm^–1^ at around 68 min. Raman modes in this range are associated with a C–S bond.^75^ A possible formation pathway of a carbon–sulfur bond would involve α-pinene reactions with a sulfate radical resulting from the strong oxidative conditions.^76,77^ Other organosulfur product pathways may be likely through intermediates formed from reactions of IEPOX with sulfate.^78,79^ The explanation for the loss of this proposed C–S mode is uncertain. The bond could be part of a molecule with an ionizable functional group in the outer organic shell phase that becomes charged when exposed to HONO/HNO_3_ and dissolves into the aqueous core, where it exhibits a weaker Raman signal. It may also undergo nucleophilic attack such as by NO_3_^–^ or NO_2_^–^. There are several known reaction pathways where a nucleophile could cleave a C–S bond.^80^ NO_3_^–^ is visible only after the disappearance of the peak at 714 cm^–1^. This delayed NO_3_^–^ peak appearance contrasts sharply with the previous droplet (Figure 3), where NO_3_^–^ was promptly visible, even with the probable onset of a core–shell morphology. This could indicate that the composition of the outer organic shell may be significant in determining when aqueous NO_3_^–^ begins to form in the droplet. Another difference between the two droplets is that the core–shell morphology in the NaHSO_4_ droplet (Figure 4) is present before exposure to HONO_(g)_, while in the NaCl droplet (Figure 3) the core–shell morphology was initiated by HONO_(g)_ exposure. The order of the morphology change could influence the NO_3_^–^ formation rate as much as the outer shell composition does.

These results for organic-containing droplets, summarized in Table 3, and NO_3_^–^ formation rates plotted in Figure S9, follow the conclusion from the previous section that HONO_(g)_ uptake is accelerated in the absence of sufficient buffer to deprotonate the incoming HONO_(g)_ such that it dissolves into the bulk droplet volume. At the surface, HONO_(ads)_ is quickly oxidized to HNO_3_, which is not substantially limited by droplet pH. The presence of organic compounds does not hinder this reaction and in some cases promotes it when organic acids are present. There is evidence that a core–shell morphology of a certain composition may slow down, but not stop, the oxidation of HONO to HNO_3_. However, it cannot be ruled out that the conversion of HONO to HNO_3_ proceeds unhindered but that reactions between HONO/HNO_3_ and organic components in the outer organic shell phase may delay, suppress, or compete with the formation of NO_3_^–^ in the aqueous core phase. The AOT experiments cannot distinguish between these two possibilities. If the conversion of HONO to HNO_3_ is slowed or hindered, the outer organic phase may reduce the reactive uptake probability (up to a factor of 5) for HONO_(g)_ through the presence of compounds that react with HONO/HNO_3_ slower than the oxidation of HONO to HNO_3_ proceeds. The loss of the proposed C–S bond at 714 cm^–1^ in Figure 4 before the formation of NO_3_^–^ is observed is strong evidence that chemical reactions occur in the outer shell prior to visible NO_3_^–^ formation in the aqueous core and that only specific functional groups, if present in the outer layer, are subject to these reactions.

**Table 3:** Summary of Aqueous Inorganic Droplets with Organic Constituent Composition and Kinetics under High HONO Exposure (46+ ppm_v_)

droplet composition	comp. 1^a^ (M)	comp. 2^a^ (M)	no. of droplets	initial pH (bulk)	NO_3_^–^ formation rate (M s^-1^)	reactive uptake coefficient	HONO-induced morphology change
(NH_4_)_2_SO_4_ and 3MGA	3	1.5	2	2.47	0.015	9.4 × 10^–5^	homogenous → core–shell^b^
(NH_4_)_2_SO_4_ and GA	2	2	1	2.56	0.024	1.4 × 10^–4^	No
NaCl and α-pinene SOA	saturated	–	1	n/a	0.028	1.5 × 10^–4^	emulsion → core–shell
NaHSO_4_ and α-pinene SOA	4	–	1	n/a	0.0057	3.6 × 10^–5^	remained core–shell^c^

a Initial concentrations of the first and second components listed.

b Morphology change tentative, not confirmed by WGM analysis.

c Droplet had a core–shell morphology prior to HONO exposure.

## Discussion

These experiments are the first to examine the spontaneous oxidation of HONO in levitated aerosol microdroplets that can reveal potential droplet accelerated mechanisms and kinetics, distinguishing them from the bulk aqueous experiments conducted in previous studies. These are also the first experiments conducted in systems beyond isolated HONO, water, and air that better mimic real-world tropospheric aerosol compositions by including secondary organic aerosol from terpene oxidation and similar proxies. Nitrite was never observed in the Raman spectra of microdroplets exposed to HONO_(g)_, while the rapid formation of nitrate was observed instead. This spontaneous oxidation of NO_2_^–^ to NO_3_^–^ is not observed under an N_2_ atmosphere, suggesting that O_2_ is the primary oxidant involved, as was observed in prior bulk experiments.

Our experimental results reveal that under many reaction conditions with ppm_v_ concentrations of HONO, substantial HONO uptake onto acidic droplets is possible, not due to partitioning and dissolution but rather via the autoxidation of HONO to HNO_3_. This conversion can occur in droplets with a high ionic strength or with organic components and shell phases present. This acceleration is attributed to acid promotion of the reaction kinetics by keeping HONO in its protonated form, which likely causes it to concentrate at the interface and promotes formation of a HONO complex, such as a HONO·HONO dimer (eq R1), [HONO·O_2_] (eq R3), or protonated HONO (eq R9), that is then oxidized by O_2_ to HNO_3_ following a second-order dependence on [HONO]. Considering the likely second-order dependence on [HONO] and no apparent reaction rate difference between NaCl and NaHSO_4_ droplets, the HONO dimer is the most probable intermediate prior to oxidation by O_2_. This agrees with the conclusion reached by Damschen and Martin (eqs R1 and R2) and by Takenaka et al. (eq E5).^34,35,43^

The presence of ample buffer such as SO_4_^2–^ can decrease the HONO oxidation rate in droplets by maintaining near-neutral pH and allowing for the deprotonation of HONO and transport from the droplet interface into the bulk. Oxidation of NO_2_^–^ then proceeds through a slower mechanism likely with a first-order dependence on NO_2_^–^ where no HONO complex formation is necessary.

A moderate HONO oxidation rate decrease was observed under some core–shell morphology conditions where secondary organic aerosol forms a shell around the aqueous core and contains functional groups that may be reactive with HONO or its products. The observation that HONO oxidation still occurs readily on aqueous–organic core–shell droplets is further evidence that the reaction likely occurs at the interface involving a HONO complex and O_2_ and not dissolved/solvated reactants.

The description of HONO chemistry with surfaces including on aerosol remains poorly described in atmospheric chemical models. These findings provide a new understanding regarding the unique reaction mechanisms and accelerated kinetics enabled by aerosol droplet interfaces and may have important previously unrecognized implications for the chemical fate and lifetime of HONO, an important driver of atmospheric oxidant budgets and chemistry. Considering that atmospheric chemical models still cannot account for the observed tropospheric concentrations of HONO, a new aerosol sink of HONO would imply that there are still important missing sources of HONO in the troposphere. The photolysis of HNO_3_ in aerosols and snowpack and on surfaces has been recently proposed as a source of HONO, for example.^9,81,83^ This recycles HONO following its oxidation to HNO_3_, and our new findings of the efficient oxidation of HONO on aerosol droplet surfaces would greatly change the dynamics and partitioning over its chemical lifecycle. As droplet-accelerated chemistry is driven by interfacial chemistry, the reaction rates reported here from supermicron droplets likely represent lower limits to the rate of HONO oxidation on the surface of submicron deliquesced accumulation-mode aerosol particles. The rate on submicron droplets may be orders of magnitude higher due to the greater surface area to volume ratio.^51^

## Data Availability

Data for this research, including processed droplet data and figure data, are available at Zenodo.org at DOI: 10.5281/zenodo.12537668.

## Supporting Information Available

Additional experimental details, including a schematic and representative spectrum, and additional experimental results, including data figures (PDF)
