# Supplementary material for: Spontaneous Oxidation of Nitrous Acid to Nitric Acid in Supermicron Aqueous Droplets Is Acid-Accelerated
Source: ACS Earth Space Chem. 2025 Apr 21;9(5):1152–64. doi: 10.1021/acsearthspacechem.5c00014 (PMC12086856; doi:10.1021/acsearthspacechem.5c00014)
Supplement: Supplementary file 1 — sp5c00014_si_001.pdf [file sp5c00014_si_001.pdf]

Supplemental Information for:

Spontaneous oxidation of nitrous acid to nitric acid in supermicron aqueous droplets is acid accelerated

Luke W. Monroe,[a]† Jack W. Hall,[a] Graham M. Thornhill, [a] and Ryan C. Sullivan* [a,b]

[a]Department of Chemistry, Carnegie Mellon University, Pittsburgh, Pennsylvania 15213, United States

[b]Department of Mechanical Engineering, Carnegie Mellon University, Pittsburgh, Pennsylvania 15213, United States

†Current address: Department of Natural Science, Northwest Missouri State University, Maryville, Missouri, 64468, United States

* Corresponding author: R.C. Sullivan, rsullivan@cmu.edu

Table of contents:

S1: Description of the step-and-glue process to acquire the Raman spectrum

S2: Additional information of the HONO gas generation process

S3: Additional information of bulk and droplet-on-substrate analysis

S4: Kinetic methods to quantify sodium nitrite oxidation by oxygen and ozone

S5: Reactive uptake coefficient determination

S6: Variation in droplets under N_2 environments and likely causes

S7: On droplet size dependence, trace oxidants created at droplet interface

Figure S1: AOT Chamber design

Figure S2: Composite Raman spectrum of major modes

Figure S3: 4 M $NaNO_2$ droplet in ultrapure O_2

Figure S4: 4 M $NaNO_2$ aqueous droplet under N_2 atmosphere

Figure S5: A saturated NaCl droplet exposed to HONO

Figure S6: The WGM fit for the droplets shown in Figure 1

Figure S7: A 4 M $NaHSO_4$ exposed to HONO under N_2 atmosphere

Figure S8: A 3 M $(NH_4)_2SO_4$ droplet with 1.5 M 3-methyl glutaric acid exposed to HONO

Figure S9: NO_3^- formation rate determined for various organic-containing droplet compositions

Supplemental References

S1: Description of the step-and-glue process used to acquire the Raman spectrum. The Raman spectrograph was operated with a 1200 g/mm diffraction grating in all experiments. At times, the default spectral window was expanded by taking individual frames across a broader wavenumber range by rotating the diffraction grating and then stitching the individual frames together (referred to as step-and-glue). This process came with the cost of increased time between successive frame acquisitions which may hinder droplet fitting via WGM analysis but allowed for the incorporation of the O-H stretch of H_2O from the aqueous component and the generic hydrocarbon C-H stretch in the organic component to be monitored concurrent with observing changes in droplet chemical composition, especially in the fingerprint region.

S2: Additional information of the HONO gas generation process. The concentration of the $HONO_{(g)}$ source was found to have constant $HONO_{(g)}$ production for the first 15 minutes and slowly decreased over an hour. There was little to no release of HONO past one hour of output upon the completion of reaction with aqueous $NaNO_2$ and H_2SO_4 in the gas washer reservoir. The resulting HONO concentrations were 46 ± 13 to 140 ± 37 ppm in the chamber during the first 15 minutes of HONO exposure depending on the HONO flow rate used in the high concentration experiments. The HONO concentration was 3 to 9 ppm for low concentrations experiments.

S3: Additional information of bulk and droplet-on-substrate analysis. Bulk aqueous experiments were analyzed by redirecting the 532 nm excitation laser to a cuvette holder. Raman spectra were collected tangential to this beam path using the same spectrometer used for the AOT, with the 1200 g/mm diffraction grating.

HONO production was calculated via aqueous uptake in a pH-neutral 4 M NaCl bulk solution and monitoring any change in acidity via a pH electrode (Thermo Scientific) for two hours. pH measurements of solutions used to generate droplets were conducted to determine the bulk pH of each droplet solution prior to nebulization.

Droplets on a substrate were examined using Raman microscopy by pipetting 1 μL solution volumes onto a siliconized hydrophobic glass coverslip (Hampton Research) at the terminus of the AOT beam path on the objective at the bottom of the lower trapping chamber. The backscattered Raman signal was directed to the spectrometer on the same path as used for levitated droplets. The AOT chamber was placed over the coverslip in the chamber's base plate to maintain constant humidity and permit the use of the same HONO gas production system as is used for the tweezed droplets.

S4: Kinetic methods to quantify sodium nitrite oxidation by oxygen and ozone. The oxidation of nitrite by oxygen was previously modeled by Hunt *et al.* using:1

$$\frac{[NO_2^-]_t}{[NO_2^-]_i} = e^{-P_{O2}H'kt} \qquad (2)$$

Where P_{O2} is the partial pressure of oxygen in atm, *H'* is Henry's law constant adjusted to ionic strength in M atm^{-1}, *k* is the rate constant in M s^{-1}, and *t* is the time of the reaction in s. *H'* for oxygen was determined by using the Sechenov equation and found to be 9.86 x 10^{-4} M atm^{-1}. This kinetic equation is first presented in Smith *et al.*5 and describes the uptake of a gaseous reactant via the

loss of a condensed-phase species while accounting for the non-reactive accommodation of the gaseous reactant into the aerosol liquid phase. In the case of this equation, the reactive-diffusive length of oxygen exceeds the diameter of the droplet and as such is volume-limited and not surface-limited.

S5: Reactive uptake coefficient determination. The equation used to determine the reactive uptake coefficient is from Smith et al.[4] and takes the form:

$$\gamma = \frac{\frac{d[NO_3^-]}{dt} r4RT}{3P_{HONO}\omega}$$

Where γ is the reactive uptake coefficient, *r* is the radius of the droplet, *R* is the gas constant, *T* is temperature, P_{HONO} is the partial pressure of HONO, and ω is the root mean square speed of HONO.

S6: Variation in droplets in N_2 environments and likely causes. A subset of droplets exposed to $HONO_{(g)}$ in a pure N_2 environment do show uptake like that seen in normal atmospheric conditions containing O_2. This subset of droplet experiments was likely unintentionally exposed to oxygen due to a leak in the HONO gas washer source or at the O-ring that seals the chamber around the glass coverslip underneath the trapped droplet. These sites contain joints that are difficult to make airtight, particularly around the glass coverslip where visibility on the seal is impossible and the O-ring may shift during chamber installation.

$NaNO_2$ droplets under an N_2 environment were generally more reproducible in not showing NO_3^- formation. Almost all $NaNO_2$ droplets showed some initial oxidation after aerosolization. This oxidation would shortly cease and remain far below the oxidation extent seen in the $NaNO_2$ droplets exposed to oxygen or ozone. This short period of oxidation was due to the oxygen introduced into the chamber by the nebulizer and was quickly moved out of the chamber once a droplet was trapped. The more reliable nature of the $NaNO_2$ droplets that had a simpler experimental setup provides strong evidence that it is leaks in the more complicated experimental setup that led to occasional oxidation under N_2 conditions.

S7: On droplet size dependence, trace oxidants created at droplet interface. To determine if there was a droplet size dependence on these reaction kinetics and potential impact via droplet accelerated mechanisms, NaCl droplets were placed directly on the coverslip at a volume of 1 µL (a levitated droplet with a radius of 3400 nm has a volume of 0.18 pL). The observed rate of NO_3^- formation is 0.012 M s^{-1}. A similar rate is seen in the much smaller trapped droplets and provides evidence that the reaction rate is likely not size-dependent. Although we concluded that the oxidation of HONO occurs at the interface, it does not seem sensitive to the amount of surface area available under the conditions used here. The ppm levels of HONO and/or supermicron droplet sizes may obviate any surface area dependency.

Droplets-on-substrate containing high concentrations of furfuryl alcohol or terephthalic acid as radical oxidant scavengers were subjected to HONO vapor exposure, with both droplets still showing rapid NO_3^- formation. However, the degradation of the droplet Raman signal shortly after visible nitrate mode formation made rate quantification difficult in these two experiments. The rapid

degradation was likely the result of the protonation of terephthalic acid significantly reducing its solubility and causing precipitation for the first system, and photo-initiated reactions of furfuryl alcohol in the laser light or acid-catalyzed polymerization that formed a brown substance that would saturate the spectrograph's detector. The unhindered formation of a NO_3^- peak, in either case, is strong evidence that trace oxidant formation is not behind NO_3^- formation.

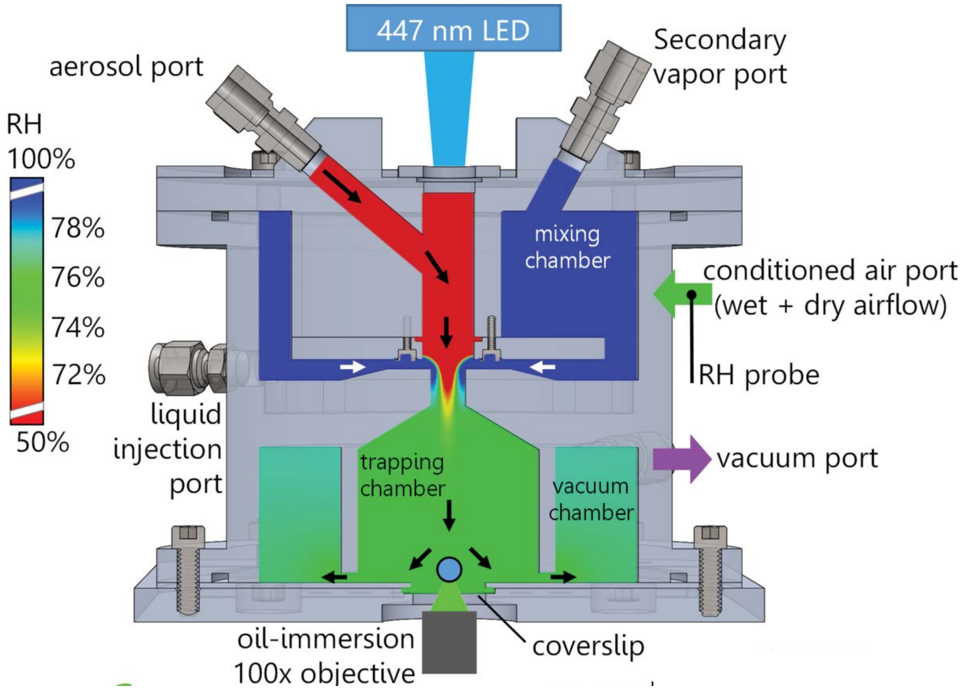

Figure S1: A SolidWorks schematic of the AOT interior and exterior design. The droplet is trapped in the bottom chamber just above the microscope coverslip. The color bar represents the computational fluid dynamics simulations for relative humidity in the chamber. Relative humidity in the trapping chamber was between 70 and 80% in all experiments in this study. Arrows indicate direction of air flows. The 447 nm LED light was not used during Raman collection to remove background signal, particularly in the fingerprint region. Figure from Gorkowski, et al.[3] Copyright 2016 American Association for Aerosol Research.

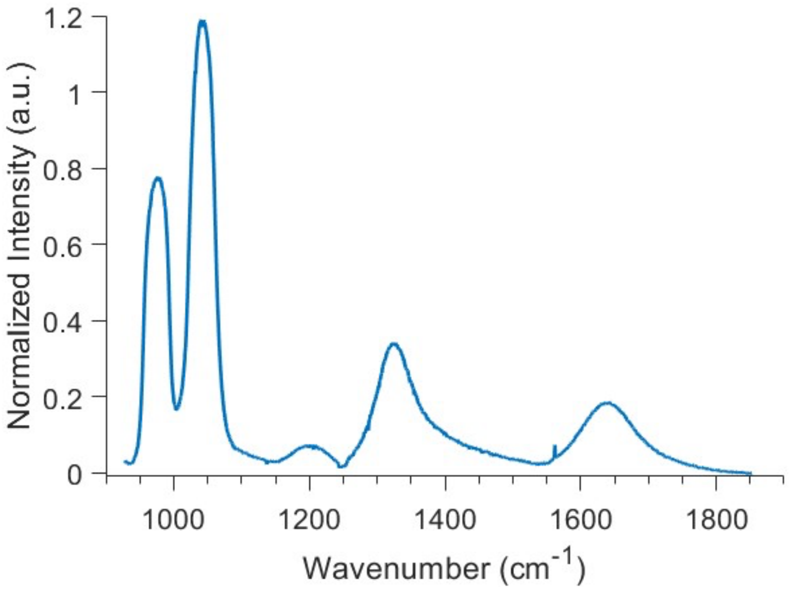

Figure S2: A composite bulk Raman frame showing relevant Raman modes used for quantification and identification of droplet chemistry. Not all modes were present at the same pH and frame made by stitching relevant modes from two separate samples. Top 4 largest peak identification from left-to-right: SO_4^{2-} (978 cm^{-1}), NO_3^-/HSO_4^- (1043 cm^{-1}), NO_2^- (1325 cm^{-1}), H_2O (1639 cm^{-1}). NO_3^- (1035 cm^{-1}) and HSO_4^- (1051 cm^{-1}) can be distinguished from each other by examining how these Raman modes change over time, as shown in Fig. 1. The fifth peak at 1200 cm^{-1} is too weak for reliable measurement in a tweezed droplet. The sharp signal at 1562 cm^{-1} is likely from a cosmic ray. All spectra were normalized to the H_2O mode peak height.

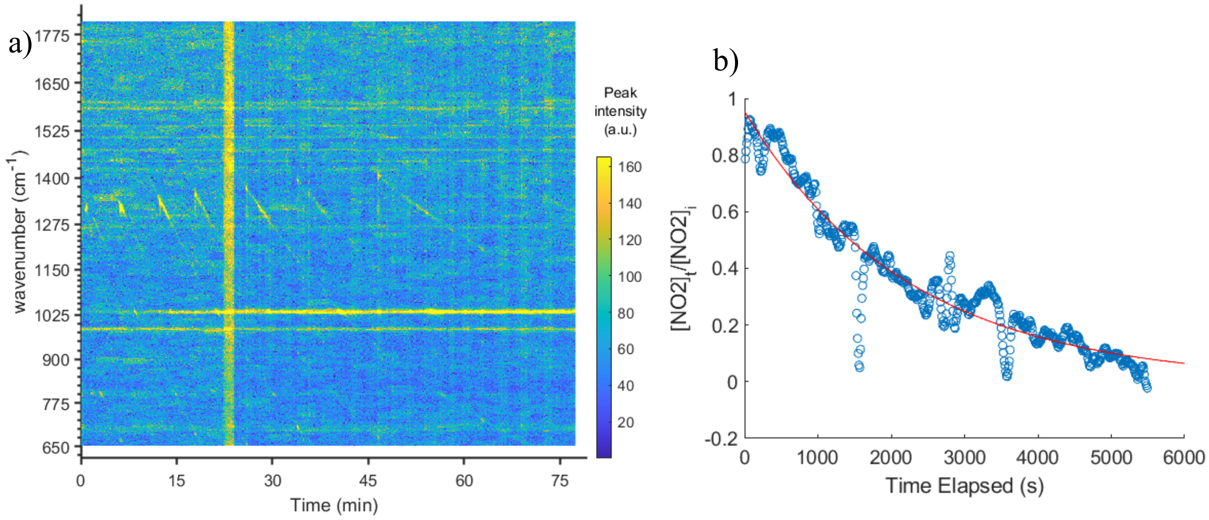

Figure S3: a) A 4 M $NaNO_2$ droplet in 100% ultrapure O_2 gas. Nitrate peak formation is visible at 1035 cm^{-1}. The signal at 987 cm^{-1} is the background peak from the immersion oil. b) Ratio of NO_2^- concentration over initial concentration versus time. Blue circles are experimental data points from Raman signal in a). Red line indicates kinetic model fit using Eqn. (3). The sudden jumps or dips in b) at approx. 1600 s, 2800 s, and 3600 s are likely the passing of WGM's through or near the nitrate mode.

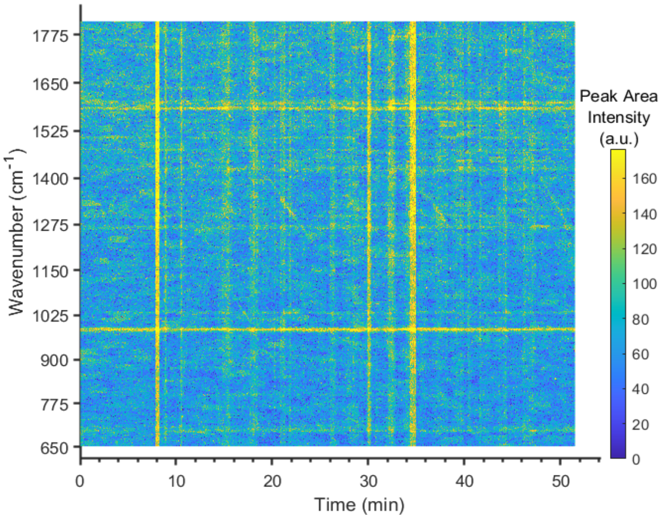

Figure S4: 4 M $NaNO_2$ aqueous droplet under N_2 atmosphere. Modes at 1000 and 1575 cm^{-1} are background interference from microscope objective immersion oil. Vertical stripes are room light interference. Nitrate from HONO oxidation would be present at 1035 cm^{-1} but is not observed.

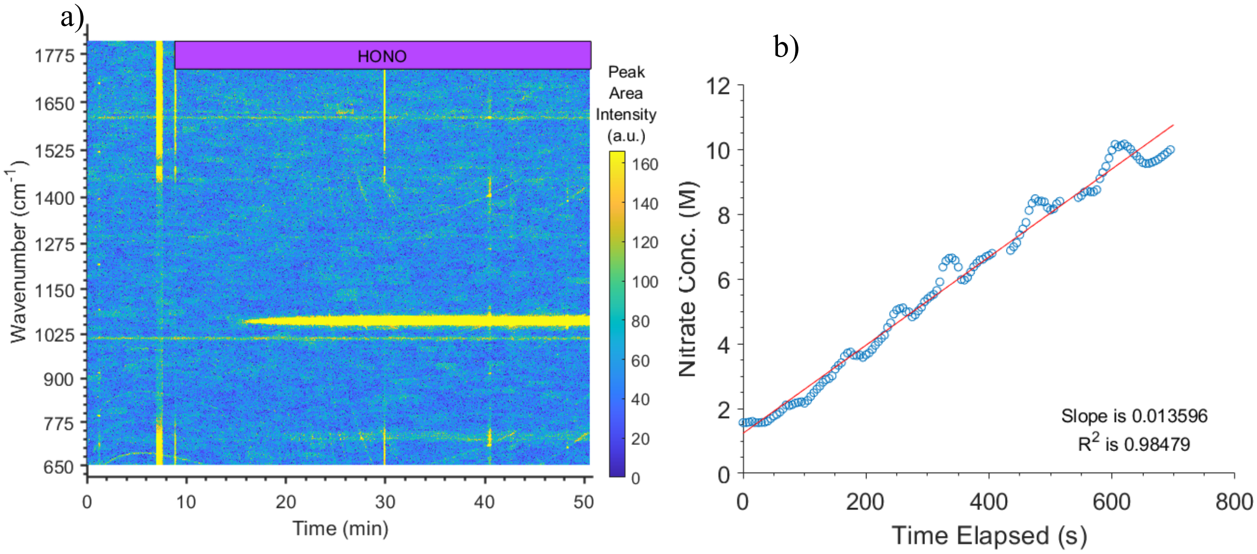

Figure S5: a) A saturated NaCl droplet exposed to HONO. Exposure period marked by a purple bar along the top. The formation of the NO_3^- Raman mode is visible at 1035 cm^{-1}. WGMs are visible at times indicating homogenous droplet morphology, though no WGM fit is possible due to their ephemeral nature. b) Nitrate concentration versus time for the droplet in a) determined from Raman spectra. Blue circles represent experimental data while the red line shows a linear regression used to determine the nitrate formation rate. Note, the zero time in b) indicates the start of peak formation of nitrate at approximately 15 minutes and is not the same zero as in a). Data before and after in b) are constant concentrations and were removed to avoid interfering with the fit quantifying peak formation.

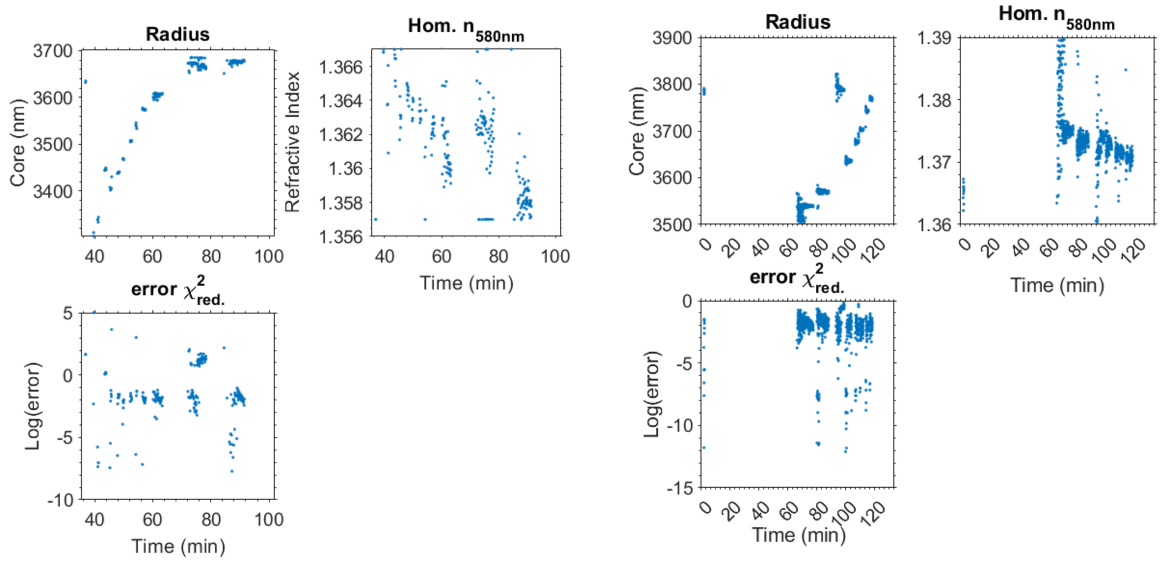

Figure S6: The WGM fit for the droplets shown in Figure 1 a) (left) and b) (right) in the main text. Left (droplet for Figure 1a) did not provide any WGM fit information before or during HONO exposure. WGM fit post-exposure indicates increasing droplet radius and decreasing refractive index at 580 nm. Fit error shows high validity for WGM fits except for a short period just prior to 80 mins. Right (droplet for Figure 1b) shows an initial measurement prior to HONO exposure and then post-exposure information. Droplet radius decreases from 3800 nm to 3550 nm during HONO exposure while refractive index increases from 1.365 to approximately 1.39. Post-exposure, size increases back to initial starting size and refractive index decreases to 1.372, likely from additional water uptake. Fit error shows valid WGM Mie model fit throughout.

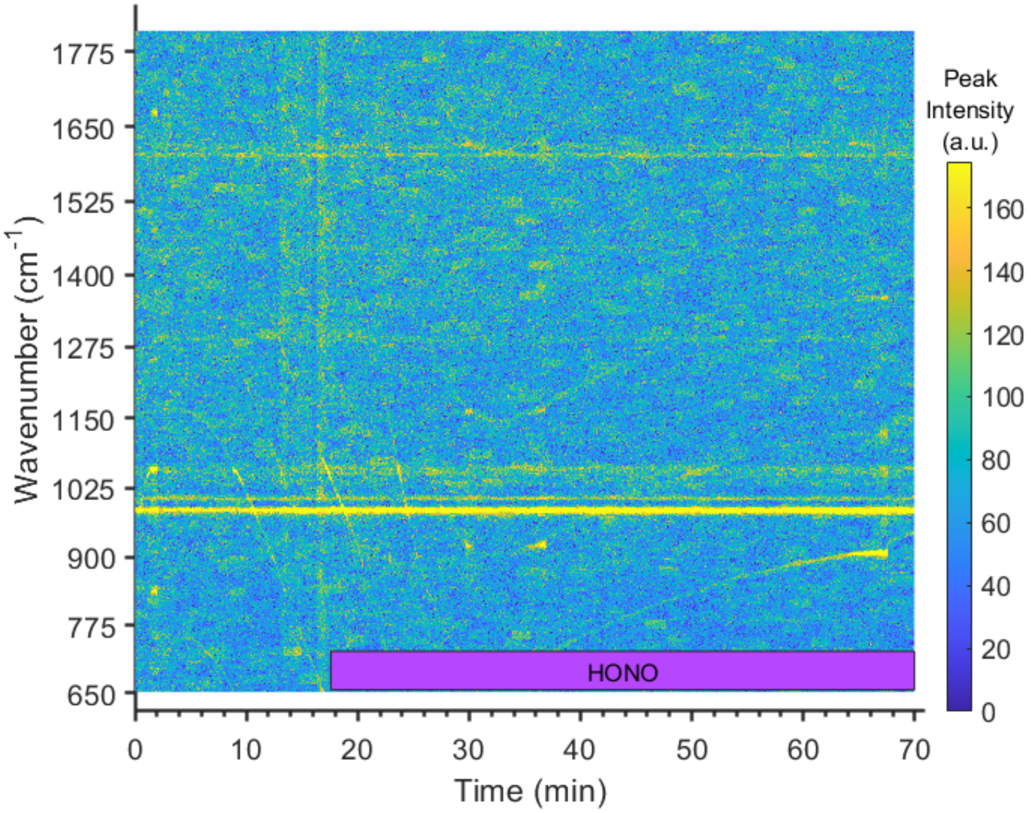

Figure S7: A 4 M $NaHSO_4$ droplet exposed to HONO under N_2 atmosphere. The purple bar on the bottom indicates the period of HONO exposure. SO_4^{2-} is visible at 980 cm^{-1} and HSO_4^- at 1051 cm^{-1}. A NO_2^- Raman peak, if present, would appear at 1345 cm^{-1}, and NO_3^- at 1035 cm^{-1}. The faint peaks at 987 cm^{-1} and 1600 cm^{-1} are background modes from the microscope objective oil.

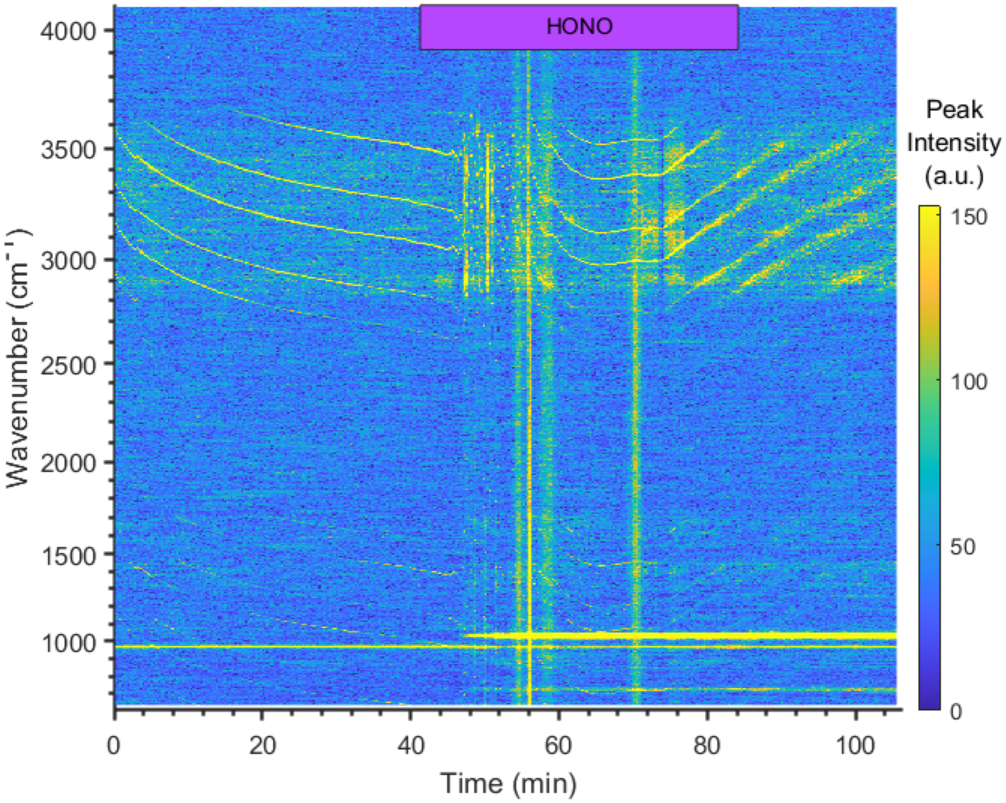

Figure S8: A 3M $(NH_4)_2SO_4$ droplet with 1.5 M 3-methyl glutaric acid exposed to HONO (purple bar, top). SO_4^{2-} loss is visible at 980 cm^{-1}. NO_3^- formation is visible at 1035 cm^{-1}. Organic C-H mode is faintly visible at 2900 cm^{-1}, and water O-H mode from 3300 to 3600 cm^{-1}. Starting at minute 48, organic carbon signals in the fingerprint region become apparent as WGMs in the O-H mode (3300-3600 cm^{-1}) demonstrating rapid droplet change. Starting at minute 80, WGMs in O-H mode become diffuse, perhaps indicating formation of a core–shell morphology which can affect retrieval of WGM Raman signal from the aqueous core.

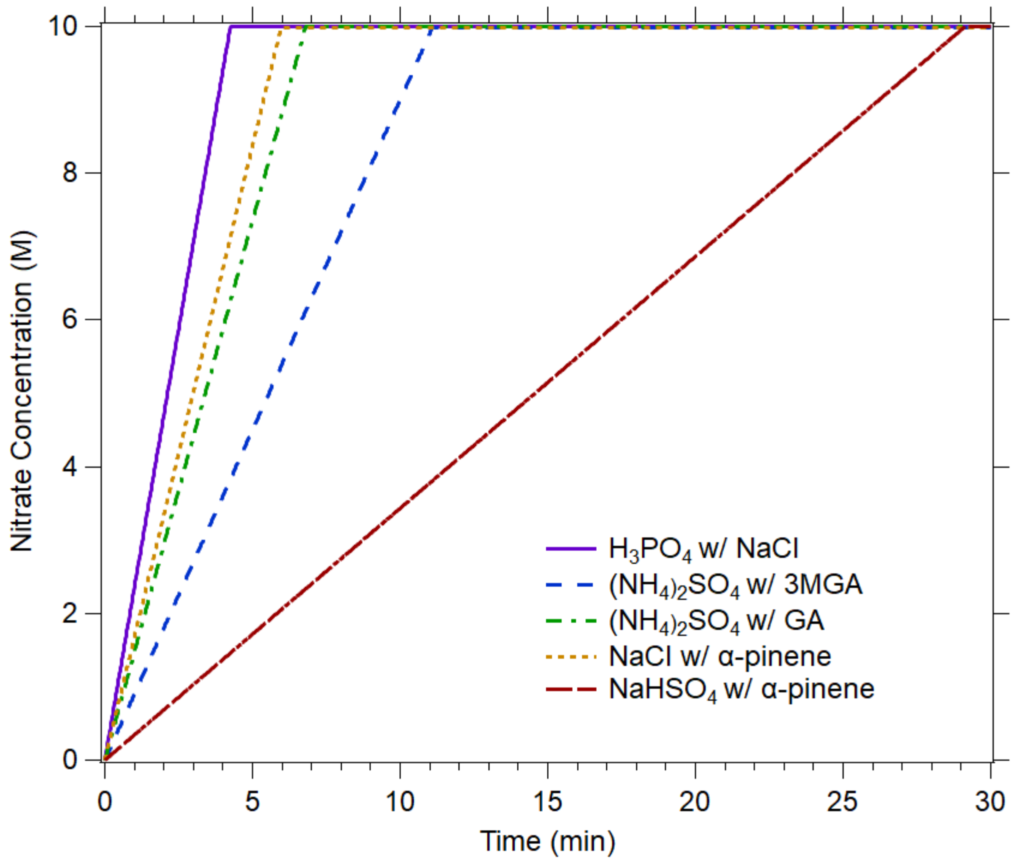

Figure S9: NO_3^- formation rate determined for various droplet organics compositions. H_3PO_4 droplet (purple) was 4 M H_3PO_4 and 1 M NaCl. $(NH_4)_2SO_4$ (3 M) with 3-methyl glutaric acid (1.5 M) is shown in blue. $(NH_4)_2SO_4$ (3 M) with glutaric acid (2 M) is in green. The seed droplets with α-pinene SOA oxidation products were saturated NaCl and 4 M $NaHSO_4$.

Supplemental References

1 O. R. Hunt, A. D. Ward and M. D. King, *Physical Chemistry Chemical Physics*, 2014, **17**, 2734–2741.

2 G. D. Smith, E. Woods, C. L. DeForest, T. Baer and R. E. Miller, *Journal of Physical Chemistry A*, 2002, **106**, 8085–8095.

3 K. Gorkowski, H. Beydoun, M. Aboff, J. S. Walker, P. Jonathan, R. C. Sullivan, K. Gorkowski, H. Beydoun, M. Aboff, J. S. Walker and P. Jonathan, *Aerosol Science and Technology*, 2016, **50**, 1327–1341.
